# Neutralizing IL-16 enhances the efficacy of targeting Aurora-A therapy in colorectal cancer with high lymphocyte infiltration through restoring anti-tumor immunity

**DOI:** 10.1038/s41419-023-06381-z

**Published:** 2024-01-30

**Authors:** Shiang-Jie Yang, Sheng-Tsung Chang, Kung-Chao Chang, Bo-Wen Lin, Kwang-Yu Chang, Yao-Wen Liu, Ming-Derg Lai, Liang-Yi Hung

**Affiliations:** 1 The Institute of Basic Medical Sciences, College of Medicine, National Cheng Kung University, Tainan, 70101 Taiwan, ROC; 2 Department of Biotechnology and Bioindustry Sciences, College of Bioscience and Biotechnology, National Cheng Kung University, Tainan, 70101 Taiwan, ROC; 3 Department of Pathology, Chi-Mei Medical Center, Tainan, 71004 Taiwan, ROC; 4 Department of Pathology, National Cheng Kung University Hospital, College of Medicine, National Cheng Kung University, Tainan, 70101 Taiwan, ROC; 5 Department of Surgery, National Cheng Kung University Hospital, College of Medicine, National Cheng Kung University, Tainan, 70101 Taiwan, ROC; 6 Department of Oncology, National Cheng Kung University Hospital, College of Medicine, National Cheng Kung University, Tainan, 70101 Taiwan, ROC; 7 National Institute of Cancer Research, National Health Research Institutes, Tainan, 70456 Taiwan, ROC; 8 Department of Pathology, Kuo General Hospital, Tainan, 70054 Taiwan, ROC; 9 Department of Pharmacology, College of Medicine, National Cheng Kung University, Tainan, 70101 Taiwan, ROC; 10 University Center for Bioscience and Biotechnology, National Cheng Kung University, Tainan, 70101 Taiwan, ROC; 11 Graduate Institute of Medicine, College of Medicine, Kaohsiung Medical University, Kaohsiung, 80708 Taiwan, ROC

**Keywords:** Immunology, Cancer

## Abstract

Cancer cells can evade immune elimination by activating immunosuppressive signaling pathways in the tumor microenvironment (TME). Targeting immunosuppressive signaling pathways to promote antitumor immunity has become an attractive strategy for cancer therapy. Aurora-A is a well-known oncoprotein that plays a critical role in tumor progression, and its inhibition is considered a promising strategy for treating cancers. However, targeting Aurora-A has not yet got a breakthrough in clinical trials. Recent reports have indicated that inhibition of oncoproteins may reduce antitumor immunity, but the role of tumor-intrinsic Aurora-A in regulating antitumor immunity remains unclear. In this study, we demonstrated that in tumors with high lymphocyte infiltration (hot tumors), higher tumor-intrinsic Aurora-A expression is associated with a better prognosis in CRC patients. Mechanically, tumor-intrinsic Aurora-A promotes the cytotoxic activity of CD8^+^ T cells in immune hot CRC via negatively regulating interleukin-16 (IL-16), and the upregulation of IL-16 may impair the therapeutic effect of Aurora-A inhibition. Consequently, combination treatment with IL-16 neutralization improves the therapeutic response to Aurora-A inhibitors in immune hot CRC tumors. Our study provides evidence that tumor-intrinsic Aurora-A contributes to anti-tumor immunity depending on the status of lymphocyte infiltration, highlighting the importance of considering this aspect in cancer therapy targeting Aurora-A. Importantly, our results suggest that combining Aurora-A inhibitors with IL-16-neutralizing antibodies may represent a novel and effective approach for cancer therapy, particularly in tumors with high levels of lymphocyte infiltration.

## Introduction

The tumor microenvironment (TME) is characterized by the complex composition of different cell types that support tumor growth, metastasis, and recurrence (ref. [1]). In the TME, cancer cells shape an immunosuppressive condition to escape immune surveillance by suppressing the activity of effector cells or recruiting immunosuppressive cells. For instance, cancer cells can utilize immune checkpoints and secrete immunosuppressive cytokines to suppress the infiltration and inhibit the activity of T cells (ref. [2]). Therefore, in the TME, the effector cells are less activated and can express higher levels of immune checkpoint proteins, such as PD-1 (ref. [3, 4]). Thus, restoration of anti-tumor immunity or targeting immunosuppressive factors in the TME has been considered a promising immunotherapeutic strategy for cancer treatment.

For immunotherapy, the status of lymphocyte infiltration is a key factor determining the therapeutic efficacy in cancer patients. Recently, some reports have shown that a high percentage of tumor-infiltrating lymphocytes is associated with improved survival outcomes in patients who received therapeutic interventions, including immunotherapy (ref. [5–8]). Thus, assessing the status of lymphocyte infiltration can improve the therapeutic response to cancer therapy. The infiltration level of CD3^+^ or CD8^+^ cells classifies tumors as “immune hot” (high-infiltrated) or “immune cold” (non-infiltrated) (ref. [9–11]), and it was reported that the status of tumor-infiltrating lymphocytes is a better prognostic index than the TNM system for predicting survival outcome of CRC patients (ref. [12, 13]). A growing body of research suggests that oncoproteins can influence tumor immunity and immunotherapy (ref. [14–16]). Moreover, several studies have shown that inhibiting oncoproteins, such as c-MET, reduces antitumor immunity by promoting PD-L1 expression in various cancers (ref. [17, 18]). Therefore, it is crucial to clarify the impact of tumor-intrinsic oncoproteins on tumor immunity; through investigation of the complex intercellular communications occurring between tumor cells and the TME is important in elucidating the underlying mechanisms that drive the CRC pathogenesis and in identifying prospective therapeutic targets.

Aurora-A is a serine/threonine kinase that plays a vital role in cell mitosis and chromosome stability (ref. [19]). Previous studies have demonstrated that cancer patients with poor prognosis have higher expression levels of Aurora-A in tumor cells (ref. [20–22]). Additionally, it has been reported that Aurora-A can promote tumor progression by facilitating cell proliferation, survival, and angiogenesis by activating β-catenin-regulated signaling (ref. [23]). Furthermore, Aurora-A can increase cell viability by suppressing cell cycle arrest and cell death by the inhibition of p53 function (ref. [24, 25]); downregulation of Aurora-A induces cellular apoptosis by increasing the levels of pro-apoptotic proteins, such as cleaved caspase-3, in human cancer cells (ref. [26]). Therefore, Aurora-A inhibition is a promising antitumor strategy in cancer therapy (ref. [22, 27]). However, targeting Aurora-A has not gotten a breakthrough in cancer therapy. Therefore, it is critical to investigate the impact of targeting Aurora-A on anti-tumor immunity and assess how tumor-lymphocyte infiltration status influences the therapeutic efficacy of targeting Aurora-A.

In this study, we investigated the effects of tumor-intrinsic Aurora-A on the regulation of tumor immunity. We evaluated the association between Aurora-A expression levels and survival outcomes in tumors with various levels of lymphocyte infiltration. Our findings revealed that the correlation between Aurora-A expression and survival outcome in CRC patients depends on the status of tumor-infiltrating lymphocytes. Inhibition of tumor-intrinsic Aurora-A reduces anti-tumor immunity by upregulating IL-16 in CRC. Combinational treatment of IL-16 neutralizing antibodies improves the anti-tumor effect of Aurora-A inhibitor. In conclusion, combining an Aurora-A inhibitor with IL-16 neutralization may represent a promising strategy for CRC patients with high tumor-intrinsic Aurora-A and tumor-infiltrating lymphocytes.

## Results

### Tumor-intrinsic Aurora-A may play diverse roles in tumor progression depending on lymphocyte infiltration status

**Fig. 1:**
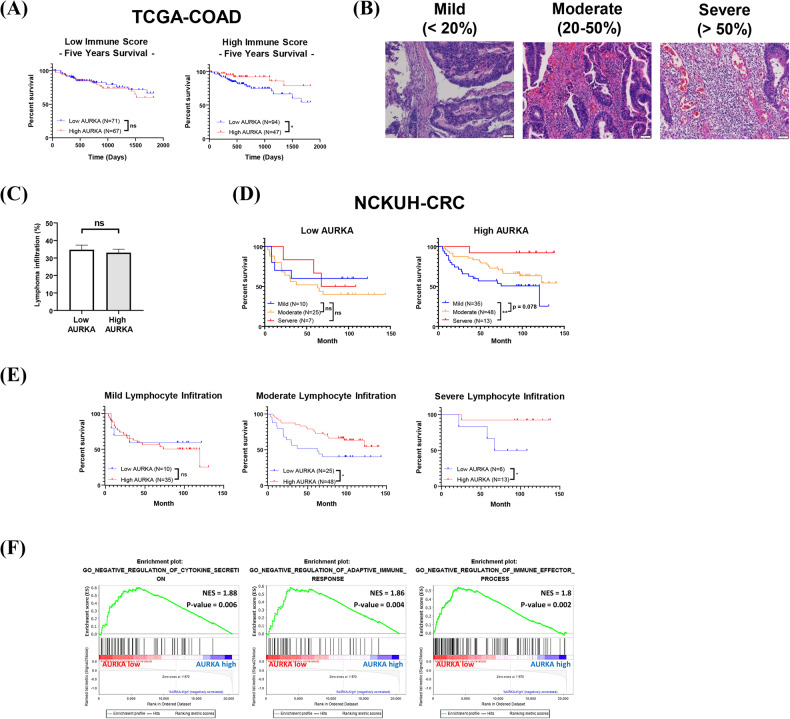
Tumor-intrinsic Aurora-A has different effects on survival rate, which depend on immune cell infiltration. **A** The overall survival rates of colon adenocarcinoma cancer (COAD) patients from the TCGA cohort were analyzed by high (red) and low (blue) expressions of Aurora-A with low (left) or high (right) immune scores in CRC. Immune scores in the TCGA COAD dataset were analyzed by xCell. KM-plot is used for statistical analysis. *, *p*-value < 0.05; ns, no significance. **B−E** CRC patients from the NCKUH cohort were enrolled for analysis. CRC patients were divided into mild, moderate, and severe lymphatic infiltration groups based on the percentage of tumor-infiltrating lymphocytes according to the H&E staining (**B**). The percentage of tumor-infiltrating lymphocytes in high and low Aurora-A (AURKA) expressed CRC is shown (**C**). The overall survival rates of CRC patients in severe (red), moderate (orange), and mild (blue) tumor-infiltrating lymphocytes with low (left) or high (right) tumor-intrinsic Aurora-A (AURKA) expression are shown. KM-plot is used for statistical analysis (**D**). **, *p*-value < 0.01. The overall survival rates of CRC patients with high (red) and low (blue) expression of Aurora-A (AURKA) in CRC patients having mild, moderate, or severe lymphocyte infiltration are shown. KM-plot is used for statistical analysis (E). *, *p*-value < 0.05. **F** Using the TCGA COAD dataset, patients are delineated into groups having either high (blue) or low (red) expression levels of Aurora-A (AURKA). The immune response signaling pathway was analyzed by gene set enrichment analysis (GSEA).

Several studies have shown that inhibition of oncoproteins, such as c-MET, may reduce antitumor immunity in various cancers (ref. [17, 18]). Since patients with high lymphocyte infiltration in the TME have a better prognosis (ref. [5, 6]), we investigated the effect of tumor-intrinsic Aurora-A on antitumor immunity in TMEs with different levels of lymphocyte infiltration. First, we performed bioinformatics analysis using TCGA datasets. We found that cancer tissues with lower immune cell infiltration (referred to as “cold tumors” (ref. [11])) and high tumor-intrinsic Aurora-A expression were associated with a poor prognosis. Conversely, cancer tissues with higher immune cell infiltration (referred to as “hot tumors” (ref. [11])) and high tumor-intrinsic Aurora-A expression showed a better prognosis (Supplementary Table S2). To validate these findings, we divided the TCGA COAD samples into high- and low-immune-infiltration groups and analyzed their overall survival rates. The results showed that patients with high immune cell infiltration and high tumor-intrinsic Aurora-A expressed CRC had a better prognosis (Fig. 1A). Furthermore, we analyzed a CRC cohort from National Cheng Kung University Hospital (NCKUH), dividing the CRC samples into mild-, moderate-, and severe-lymphocyte-infiltration groups (Fig. 1B). The expression level of Aurora-A in CRC was determined by IHC (ref. [28]). Interestingly, we found no correlation between the lymphocyte infiltration percentages and the expression levels of Aurora-A in CRC (Fig. 1C). Previous studies have demonstrated that a high level of lymphocyte infiltration in tumors is associated with a better prognosis (ref. [5–8]). However, this phenotype is exclusively observed in CRC patients with high tumor-intrinsic Aurora-A (Fig. 1D). Surprisingly, we observed that CRC with Aurora-A overexpression was associated with a better prognosis in patients with moderate and high lymphocyte infiltration (Fig. 1E).

Next, we investigated whether overexpressed Aurora-A can enhance antitumor immunity in high lymphocyte-infiltrated tumors. We conducted gene set enrichment analysis (GSEA) to identify the enriched signaling pathways in tumors with different levels of Aurora-A expression using the TCGA dataset. The results demonstrated that negative regulation of immune response signaling was enhanced in tumors exhibiting low Aurora-A expression (Fig. 1F). This finding implies that in CRC patients with reduced Aurora-A expression in the tumors, anti-tumor immunity may be more significantly suppressed. These results suggest that overexpression of Aurora-A in high lymphocyte-infiltrated CRC may play an opposite role in patients’ prognosis, depending on the regulation of immune response in the TME.

### Aurora-A promotes antitumor immunity by enhancing the infiltration and activity of CD8^+^ T cells in hot tumors

**Fig. 2:**
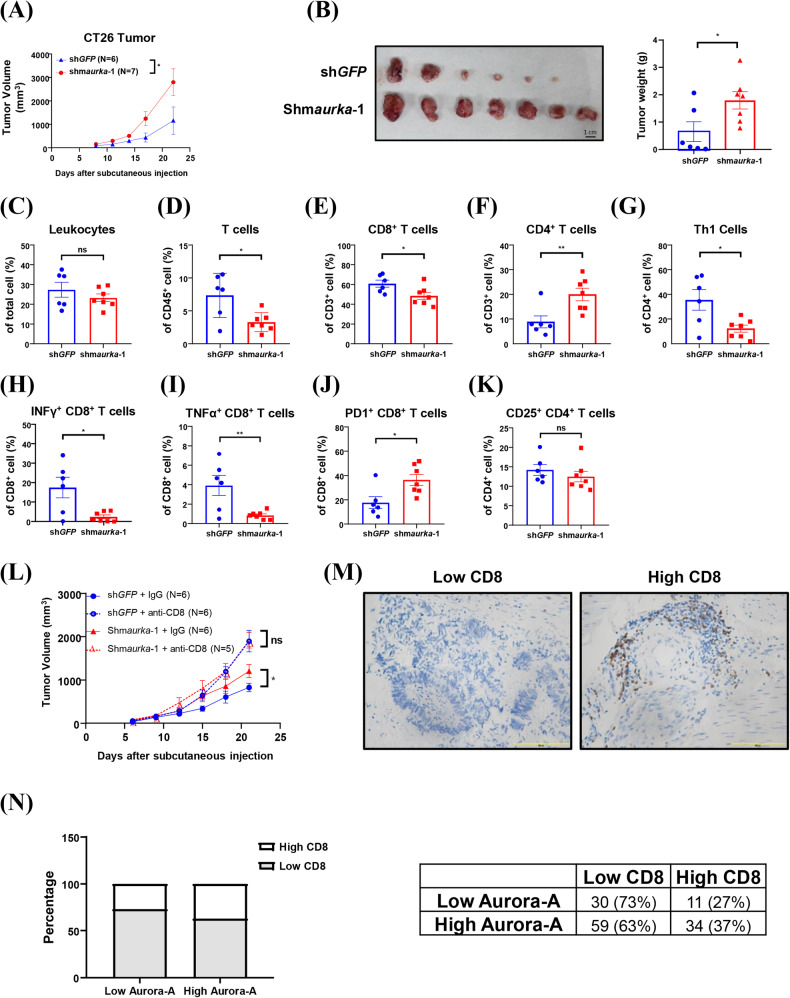
Knockdown of Aurora-A promotes tumor growth via inhibiting CD8^+^ T cell activity in immunocompetent mice. The growth of CT26-derived tumors was evaluated by the subcutaneous injection animal model in Balb/c mice. CT26 cells were infected with lenti-sh*GFP* and lenti-shm*aurka*-1 and maintained at least three passages before subcutaneous injection. **A** Tumor volume was measured every three days. Two-way ANOVA is used for statistical analysis. Data are shown as mean ± SEM; *n* = 6 or 7. *p-value < 0.05. **B** Mice were sacrificed, and tumor weight was measured on day 22 of post-subcutaneous injection. **C** Tumor-infiltrating leukocytes, (**D**) T cells, (**E**) CD8^+^ T cells, (**F**) CD4^+^ T cells, (**G**) Th1 cells (IFNγ^+^ CD4^+^ T cells), (**H**) IFNγ^+^ CD8^+^ T cells, (**I**) TNF*a*^+^ CD8^+^ T cells, (**J**) PD1^+^ CD8^+^ T cells, and (**K**) CD25^+^ CD4^+^ T cells were measured by flow cytometry. Student’s *t*-test is used for statistical analysis. Data are shown as mean ± SEM; *n* = 6 or 7. *p-value < 0.05; **p-value < 0.01. **L** Lenti-sh*GFP*-infected and lenti**-**shm*aurka*-1-infected CT26 cells were subcutaneously injected into Balb/c mice. Intraperitoneal injection of IgG or anti-CD8 antibodies was performed on day 6, day 11, and 16 post-subcutaneous injection. Tumor volume was measured every three days. Two-way ANOVA is used for statistical analysis. Data are shown as mean ± SEM; *n* = 5 or 6. *p-value < 0.05. **M**, **N** CRC patients from the NCKU cohort were enrolled for analysis. CRC tissues were divided into low and high CD8 infiltration groups based on the percentage of tumor-infiltrating CD8^+^ cells. **M** The images of high (right) or low (left) tumor-infiltrating CD8^+^ cells in CRC tissues are shown. **N** The percentage of high or low tumor-infiltrating CD8^+^ cells in CRC tumors with high or low Aurora-A expression.

According to a previous study, tumors formed from CT26 and MC38 cells were identified as “immune hot” (ref. [29, 30]) and “intermediate” tumors (ref. [29, 30]). To investigate whether Aurora-A can influence tumor growth by regulating immune cell populations, a subcutaneous tumor injection animal model was performed in immunodeficient mice and immunocompetent mice using CT26 and MC38 cells. The results showed that Aurora-A knockdown in CT26 and MC38 cells did not influence tumor growth in immunodeficient mice (Supplementary Figure S1). In contrast, Aurora-A knockdown promoted the growth of CT26-derived tumors and MC38-derived tumors in immunocompetent mice (Fig. 2A, B, S2A, B, and S3A, B). To confirm the knockdown efficacy, the expression of Aurora-A in those tumors was determined by western blot analysis (Supplementary Figures S6A–D). To identify the effects of tumor-intrinsic Aurora-A in regulating immune cell populations in the TME, we analyzed overall leukocytes, T cells, CD8^+^ T cells, and CD4^+^ T cells in Aurora-A knockdown CT26-derived tumors. We found that there was no difference in overall leukocyte infiltration (Fig. 2C and S2C), but the infiltrated T cells and CD8^+^ T cells (Fig. 2D, E and S2D, E) were decreased, and CD4^+^ T cells were increased in Aurora-A knockdown CT26-derived tumors (Fig. 2F and S2F). Infiltrating T cells, CD8^+^ T cells and CD4^+^ T cells in shGFP-infected and shm*aurka*-infected CT26-derived tumors were evaluated by IHC (Supplementary Figures S4A, B). To identify whether the knockdown of Aurora-A can influence the cytotoxic activity of tumor-infiltrating T cells, we analyzed IFNγ^+^/CD4^+^, IFNγ^+^/CD8^+^, and TNFα^+^/CD8^+^ T cell populations in tumors. The results showed that knockdown of Aurora-A reduced Th1 (IFNγ^+^/CD4^+^) (Fig. 2G and S2G) and cytotoxic CD8^+^ T (IFNγ^+^/CD8^+^ & TNFα^+^/CD8^+^) cell levels (Fig. 2H, I and S2H, I) in the TMEs of CT26-derived tumors. It is well-recognized that exhausted T cells are frequently observed in the TMEs, and this phenomenon is correlated with PD-1 high/CD8^+^ T cells (ref. [31]). Those exhausted T cells contribute to immune evasion due to reduced cytokine production and impaired cytotoxic activity (ref. [32]). Importantly, our result revealed that the proportion of PD-1^+^/CD8^+^ T cells was significantly increased in Aurora-A knockdown CT26-derived tumors (Fig. 2J and S2J). However, the CD4^+^/CD25^+^ regulatory T cells showed no difference between shGFP-infected and shm*aurka*-infected CT26-derived tumors (Fig. 2K and S2K).

Next, we aimed to investigate whether Aurora-A knockdown promotes tumor growth by inhibiting CD8^+^ T-cell cytotoxicity. To assess this, an allogeneic subcutaneous injection animal model with CD8^+^ T cell depletion was performed using anti-CD8 antibodies. As expected, the expression level of Aurora-A did not affect tumor growth after CD8^+^ T cells were depleted (Fig. 2L). Additionally, to validate whether Aurora-A expression positively correlates with CD8 infiltration in clinical samples, we analyzed Aurora-A expression and CD8 infiltration in CRC. Tumors were divided into low or high CD8 infiltration groups (Fig. 2M), and the results showed that tumors with high Aurora-A expression displayed higher CD8 infiltration (Fig. 2N). These findings suggest that tumor-intrinsic Aurora-A may promote antitumor immunity by enhancing the infiltration and activation of CD8^+^ T cells in immune hot tumors. To further validate this hypothesis, 4T1 cells, known for their tendency to form immune cold tumors (ref. [33]), were used to study the effect of Aurora-A in antitumor immunity. The results indicated that the knockdown of Aurora-A inhibited the growth of 4T1-derived tumors in immunodeficient mice (Supplementary Fig. S5A–C) and slightly reduced tumor growth in immunocompetent mice (Supplementary Fig. S5D, E). The decreased expression of Aurora-A in tumors was confirmed by western blot analysis (Supplementary Fig. S6E, F). Analysis of tumor-infiltrating leukocytes, T cells, CD8^+^ T cells, CD4^+^ T cells, Th1 cells, and cytotoxic T cells revealed no significant differences between shGFP-infected and shm*aurka*-infected 4T1-derived tumors (Supplementary Fig. S5F, S5N). These results suggest that the knockdown of Aurora-A may suppress antitumor immunity by enhancing immunosuppressive signaling pathways in tumors within immune hot TMEs.

### Aurora-A negatively regulates IL-16 expression

**Fig. 3:**
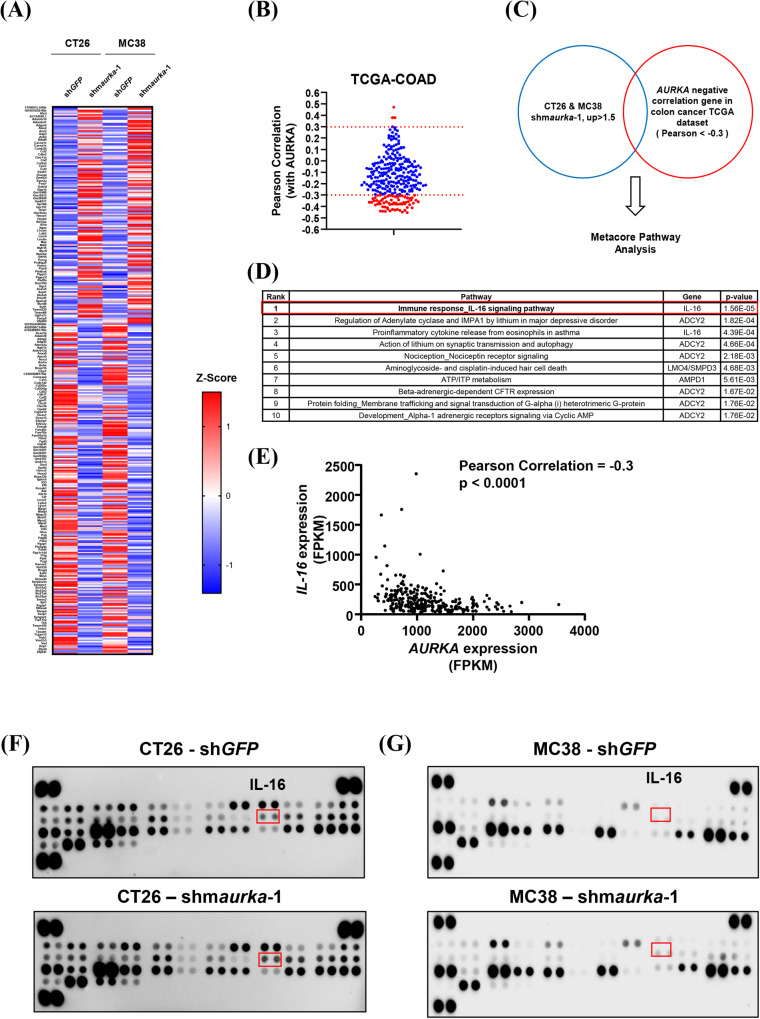
The interleukin-16 pathway is upregulated in Aurora-A knockdown CRC cells. **A** Heat map analysis shows the upregulated genes with fold change >1.5 and the downregulated genes with fold change <0.6 in both CT26 (lenti-shm*aurka*-1/lenti-sh*GFP*) and MC38 (lenti-shm*aurka*-1/lenti-sh*GFP*). **B** Correlation between potential genes and Aurora-A was analyzed using the TCGA COAD dataset by Pearson correlation. **C** Potential genes negatively regulated by Aurora-A were analyzed. The blue circle represents the upregulated genes with fold change >1.5 in both CT26 (lenti-shm*aurka*-1/lenti-sh*GFP*) and MC38 (lenti-shm*aurka*-1/lenti-sh*GFP*) (from Fig. 3B), and the red circle represents the negatively correlated genes with *AURKA* in the TCGA COAD dataset (Pearson < −0.3 from Fig. 3B). **D** MetaCore analysis was applied to genes negatively regulated by Aurora-A. **E** The correlation between *IL-16* mRNA and *Aurora-A* mRNA expression was analyzed by Pearson correlation using the TCGA COAD dataset. The Pearson correlation coefficient (r) is −0.3, and the *p*-value < 0.0001. **F**, **G** Cytokine array analyses of Aurora-A knockdown CT26 and MC38 cells are shown.

**Fig. 4:**
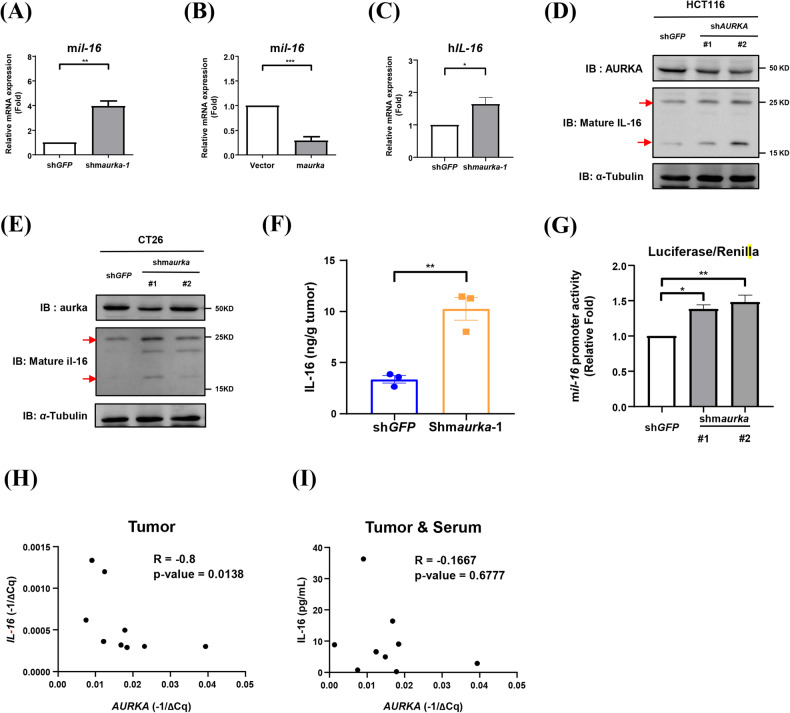
Interleukin-16 is upregulated in *AURKA* knockdown CRC cells. The expression of m*il-16* mRNA was analyzed by RT-qPCR in lenti-sh*GFP*-infected and lenti-shm*aurka*-1-infected CT26 cells (**A**), and in lenti-vector-infected and lenti-m*aurka*-infected CT26 cells (**B**). **C** The expression of *IL-16* mRNA in lenti-sh*GFP*-infected and lenti-sh*AURKA*-1-infected HCT116 cells was analyzed by RT-qPCR. Student’s *t*-test is used for statistical analysis. Data are shown as mean ± SEM; *n* = 3. *p-value < 0.05; **p-value < 0.01; ***p-value < 0.001. **D** Western blot analysis showed the expression of mature-human IL-16 protein (red arrows) in lenti-sh*GFP*-infected, lenti-sh*AURKA*-1-infected, and lenti-sh*AURKA*-2-infected HCT116 cells. **E** The expression of mature-mil**-**16 proteins in lenti-sh*GFP-*infected, lenti-shm*aurka*-1 (#1)*-*infected, and lenti-shm*aurka*-2 (#2)-infected CT26 cells (red arrows) are shown. **F** CT26-derived tumors were homogenized, and the tumor supernatants were collected to analyze the secretion of IL-16 by ELISA. Student’s *t*-test is used for statistical analysis. Data are shown as mean ± SEM; *n* = 3. *p-value < 0.05. **G** pGL3-m*il-16*-*p*romoter was transiently transfected into lenti-*GFP*-infected, lenti-shm*aurka*-1-infected and lenti-shm*aurka*-2-infected CT26 cells. The promoter activity of m*il-16* was analyzed by reporter assay. Student*’*s *t*-test is used for statistical analysis. Da*t*a are shown as mean ± SEM; *n* = 3. *p-value < 0.05; **p-value < 0.01. **H** The correlation between *IL-16* mRNA and *AURKA* mRNA in CRC is shown**. I** The correlation between serum IL-16 and *AURKA* mRNA in CRC is shown. The expression of serum IL-16 was analyzed by ELISA in CRC patients. *AURKA* mRNA was analyzed by RT-qPCR. Pearson correlation is used for statistical analysis.

To dissect the molecular mechanism of how tumor-intrinsic Aurora-A affects the function of surrounding immune cells, sh*GFP*-infected and shm*aurka*-infected CT26 and MC38 cells were collected to perform the RNA-seq analysis (Fig. 3A). The human homologs of the potential genes obtained from the RNA-seq were analyzed for their correlation with Aurora-A using the TCGA COAD dataset (Fig. 3B). Genes that exhibited more than 1.5-fold up-regulation in Aurora-A knockdown CT26 and MC38 cells, as well as genes showing a negative correlation with Aurora-A (Pearson value < −0.3) in the TCGA dataset, were selected for functional analysis using MetaCore. The results suggest that the IL-16-mediated immune response pathway is the top pathway exhibiting a negative correlation with Aurora-A expression. (Fig. 3C, D). This finding indicates a potential regulatory role of Aurora-A in modulating the IL-16-mediated immune response. Bioinformatics analysis using the TCGA dataset further confirmed the upregulation of IL-16 in lower Aurora-A-expressed CRC (Fig. 3E). Furthermore, a cytokine array confirmed the increased secretion of IL-16 in culture media collected from Aurora-A knockdown CT26 and MC38 cells (Figs. 3F, G and S7A, B). The cell model showed that m*il-16* mRNA is up-regulated in shm*aurka*-infected CT26 cells and vice versa (Fig. 4A, B), and the expression of *IL-16* mRNA was increased in Aurora-A knockdown HCT116 cells (Fig. 4C). Western blot analysis showed that the knockdown of Aurora-A increases the expression of mature IL-16 in both human and mouse CRC cells (Fig. 4D, E). Additionally, the secreted IL-16 was increased in the tumors derived from Aurora-A knockdown CT26 cells as well (Fig. 4F). Reporter assays demonstrated that m*il-16* is transcriptionally regulated by Aurora-A (Fig. 4G). The negative correlation between IL-16 and Aurora-A was further evaluated in an NCKUH human CRC cohort. As expected, the expression of *Aurora-A* mRNA was negatively correlated with that of *IL-16* mRNA in CRC (Fig. 4H); surprisingly, there was no correlation between the tumor-intrinsic *Aurora-A* mRNA level and the serum IL-16 level (Fig. 4I). This result implies that tumor-intrinsic Aurora-A may only influence the expression of IL-16 in the tumor microenvironment. These results suggest that tumor-intrinsic Aurora-A may only negatively regulate the expression and secretion of IL-16 in the TME.

**Fig. 5:**
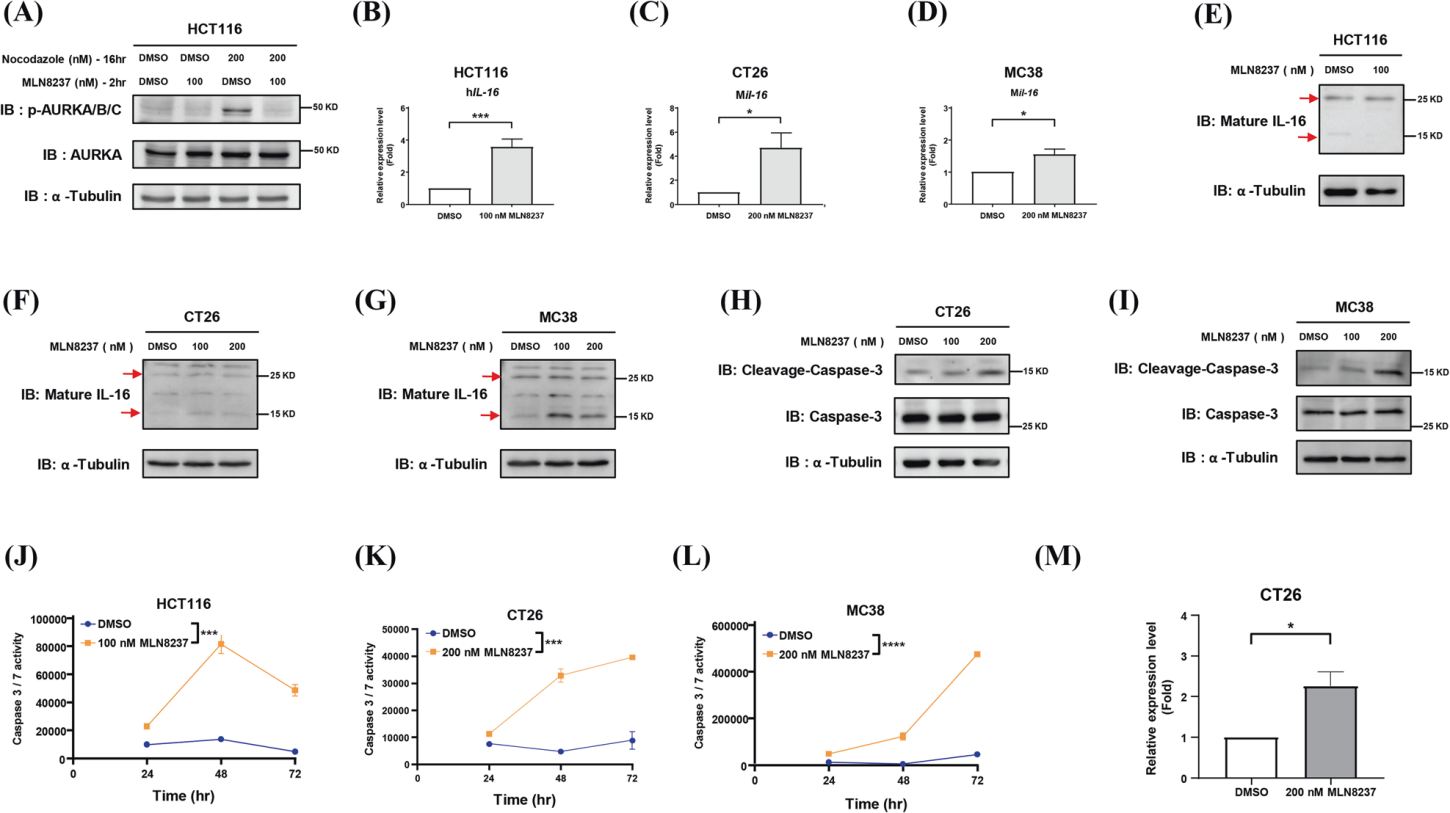
Aurora-A negatively regulating IL-16 is kinase-dependent. **A** HCT116 cells were treated with nocodazole for 16 h and then treated with MLN8237 for an additional 2 h. Total cell lysates were collected to perform western blot analysis using antibodies as indicated. DMSO was used as a no-treatment control. *IL-16* mRNA expression was analyzed by RT-qPCR in HCT116 (**B**), CT26 (**C**), and MC38 cells (**D**) after MLN8237 treatment for 48 h. Student’s *t*-test is used for statistical analysis. Data are shown as mean ± SEM; *n* = 5 (**B**, **C**) or 3 (**D**). *p-value < 0.05; ***p-value < 0.001. HCT116 (**E**), CT26 (**F**), and MC38 (**G**) cells were treated with MLN8237 for 48 h, and the expression of mature IL-16 proteins was analyzed by western blot (red arrows). CT26 (**H**, **K**), MC38 (**I**, **L**), and HCT116 (**J**) cells were treated with MLN8237 for 48 h. Cell apoptosis was analyzed by western blot (**H**, **I**) and caspase-3/7 activity (**J**−**L**). Two-way ANOVA is used for statistical analysis. Data are shown as mean ± SEM. ***p-value < 0.001; ****p-value < 0.0001. **M** CT26 cells were treated with MLN8237 for 72 h, and the secretion of IL-16 was analyzed using cultured media by ELISA. The relative fold changes of secreted IL-16 are shown. Student’s *t*-test is used for statistical analysis. Da*t*a are shown as mean ± SEM; *n* = 3. *p-value < 0.05.

The negative regulation of IL-16 by Aurora-A was further confirmed using MLN8237, an Aurora-A inhibitor. The results showed that MLN8237 effectively inhibited Aurora-A activation (Fig. 5A), and both IL-16 mRNA and protein expression were upregulated in MLN8237-treated human and mouse CRC cells (Fig. 5B–G). It has been reported that pro-IL-16 undergoes cleavage into mature IL-16 by activated caspase-3 (ref. [34]), and previous reports indicate that inhibition of Aurora-A can activate caspase-3 in cancer cells (ref. [26]). To identify whether Aurora-A inhibition promotes IL-16 maturation by activating caspase-3 activity, we measured caspase-3 activity in CRC cells treated with MLN8237. The results showed increased caspase-3 activity in MLN8237-treated CRC cells (Fig. 5H–L). In addition, inhibition of Aurora-A promoted the secretion of IL-16 in CT26 cells (Fig. 5M). These results suggest that Aurora-A negatively regulates IL-16 expression in the TME of CRC by activating caspase-3 in a kinase-dependent manner.

### Tumor-intrinsic Aurora-A promotes CD8^+^ T cell activity by decreasing IL-16 expression

**Fig. 6:**
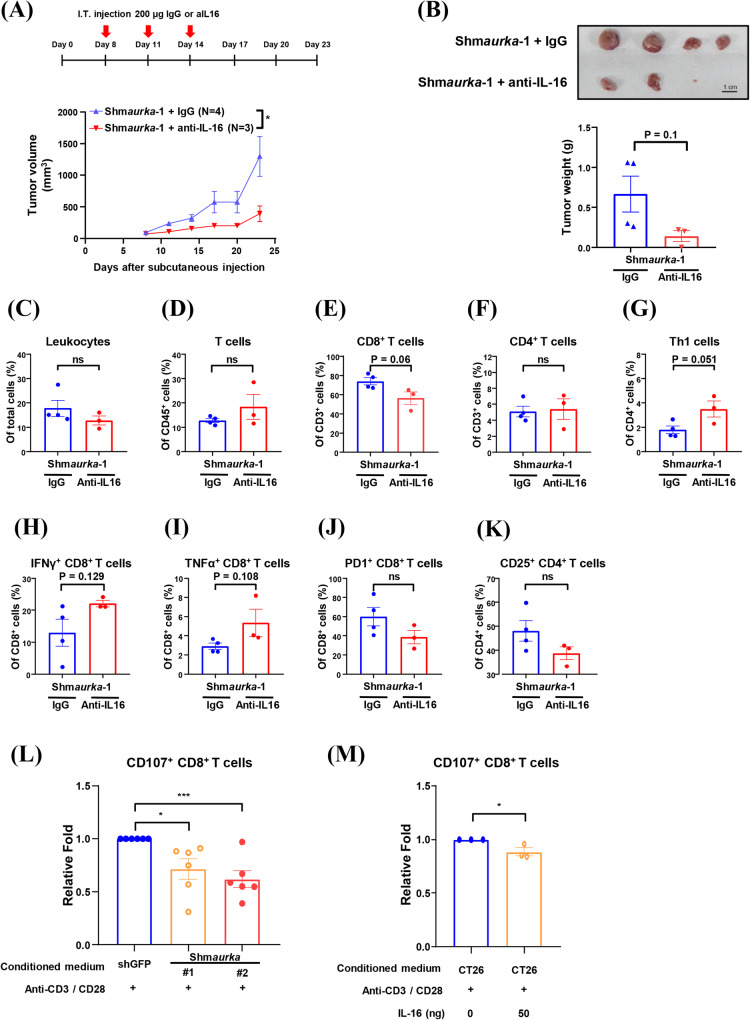
Neutralizing IL-16 reverses tumor growth and CD8^+^ T cell cytotoxicity in Aurora-A knockdown CT26 tumors. **A** A schematic illustration of antibody treatment (IgG or anti-IL16) in the allogenic animal model is shown. Lenti-sh*GFP*-infected and lanti-shm*aurka*-1-infected CT26 were subcutaneously injected into Balb/c mice; IgG or anti-IL-16 antibodies were intratumorally (IT) injected on day 8, day 11, and day 14 of post-subcutaneous injection. Tumor volume was measured every three days. Two-way ANOVA is used for statistical analysis. Data are shown as mean ± SEM; *n* = 3 or 4. *p-value < 0.05. **B−K** Mice from Fig. 6A were sacrificed on day 23 post-subcutaneous injection, and tumor weights were measured (**B**). Tumor-infiltrating leukocytes (**C**), T cells (**D**), CD8^+^ T cells (**E**), CD4^+^ T cells (**F**), Th1 cells (IFNγ^+^ CD4^+^ T cells) (**G**), IFNγ^+^ CD8^+^ T cells (**H**), TNF*a*^+^ CD8^+^ T cell (**I**), PD1^+^ CD8^+^ T cells (**J**), and CD25^+^ CD4^+^ T cells (**K**) were measured by flow cytometry. Student’s *t*-test is used for statistical analysis. Data are shown as mean ± SEM; *n* = 3 or 4. **L**, **M** CD8^+^ T cells were purified from Balb/c mice splenocytes. The purified CD8^+^ T cells were cultured in conditioned media collected from lenti-sh*GFP*-infected and lenti-sh*maurka* (#1 and #2)-infected CT26 plus anti-CD3/CD28 antibodies without (**L**) or with recombinant IL-16 (M) for 48 h. The cytotoxicity of CD8^+^ T cells in splenocytes was measured by flow cytometry using anti-CD107 antibodies. The relative fold change of CD107^+^/CD8^+^ T cells are shown. Student’s *t-test* is used for statistical analysis. Data are shown as mean ± SEM. *p-value < 0.05; **p-value < 0.01; ***p-value < 0.001; ****p-value < 0.0001.

Our results demonstrated that tumor-intrinsic Aurora-A promotes the infiltration and activity of CD8^+^ T cells in immune hot tumors (Figs. 2E and S2E). To further address the mechanisms underlying this effect, we investigated whether tumor-intrinsic Aurora-A can modulate CD8^+^ T cell cytotoxicity by regulating IL-16 production. Anti-IL-16 antibodies were used to deplete IL-16 in Aurora-A knockdown CT26-derived tumors. The results showed that anti-IL-16 antibodies attenuated tumor growth (Fig. 6A, B) and enhanced antitumor immune populations (Fig. 6C–K). Our results show that the PD1^+^/CD8^+^ T cell population is increased in Aurora-A knockdown tumors (Fig. 2J), and IL-16 neutralization slightly reduces this population (Fig. 6J). PD1-high/CD8^+^ exhausted T cells commonly express multiple inhibitory receptors (ref. [35]). Here, we investigated the correlation between IL-16 and inhibitory receptor expression. According to the data from TCGA, *IL-16* mRNA expression was positively correlated with several inhibitory checkpoint molecules in CRC (Supplementary Fig. S8A–G). To confirm whether tumor-intrinsic Aurora-A can regulate the function of CD8^+^ T cells, the cytotoxicity of CD8^+^ T cells was determined by measuring the expression of CD107 in splenocytes that were pretreated with conditioned media collected from Aurora-A knockdown CT26 cells. The results indicated that CT26 cells with decreased Aurora-A expression could inhibit CD8^+^ T cell cytotoxicity (Supplementary Fig. S8H). Further confirming this observation, the inhibition of CD8^+^ T cell activity caused by shm*aurka*-1 was rescued by shRNA-resistant Myc-m*aurka* (C-1) in a co-culture experiment (Supplementary Fig. S9A, B). Complementarily, only Aurora-A/wild type (WT)-overexpressing cells, but not the Aurora-A/kinase-dead (KD) mutant, can enhance CD8^+^ T cell activity (Supplementary Fig. S9C, D). It has been reported that mature IL-16 can preferentially induce the migration of Tregs (ref. [36]) and enhance the cytokine production of Th2 and Th17 cells in an allergic inflammation mouse model (ref. [37]). Furthermore, IL-16 depletion enhances the activities of Th1 and CD8^+^ T cells during virus infection (ref. [38]). Therefore, we turned toward assessing whether tumor-intrinsic Aurora-A can promote CD8^+^ T cell activity by regulating CD4^+^ T cells. The results showed that Aurora-A knockdown in CT26 cells could inhibit CD8^+^ T cell cytotoxicity after depleting CD4^+^ T cells in splenocytes (Supplementary Fig. S8I). We then turned to clarify whether tumor-intrinsic Aurora-A can directly influence CD8^+^ T cell activity. To address this, CD8^+^ T cells were isolated from splenocytes and incubated with a conditioned medium from Aurora-A knockdown CT26 cells. As expected, the CD8^+^ T cell cytotoxicity was directly inhibited by Aurora-A knockdown CT26 cells (Fig. 6L). The cytotoxic activity of purified CD8^+^ T cells was inhibited when cells were treated with recombinant IL-16 (Fig. 6M). These results suggest that tumor-intrinsic Aurora-A promotes the activation of CD8^+^ T cells by inhibiting IL-16 expression. Our results showed that the negative regulation of IL-16 by tumor-intrinsic Aurora-A is a universal mechanism in CRC cells (Fig. 5B–G). However, only in CRC with high lymphocyte infiltration (hot tumor), tumor-intrinsic Aurora-A can promote antitumor immunity via inhibiting the IL-16-mediated immune suppression pathway.

### IL-16 neutralization promotes the therapeutic effect of an Aurora-A inhibitor

**Fig. 7:**
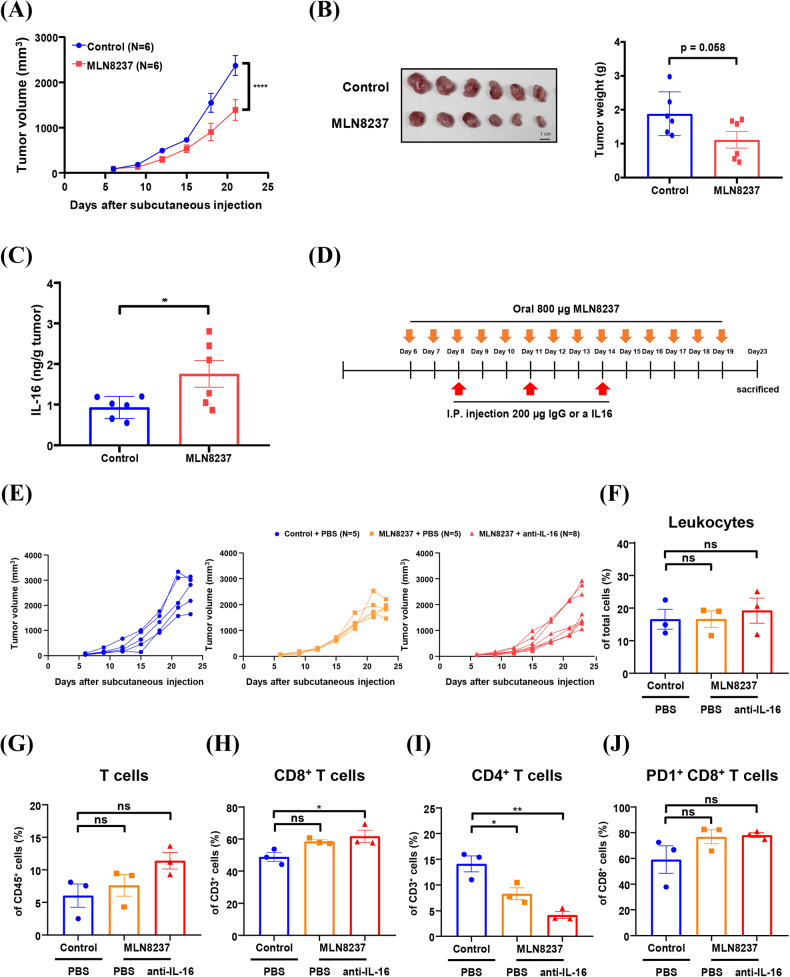
Combining Aurora-A inhibitor and anti-IL-16 antibody synergistically inhibits tumor growth in immunocompetent mice. In vivo CT26 tumor growth was evaluated by a subcutaneous mouse model in Balb/c. **A**, **B** 8-week-old Balb/c mice were subcutaneously injected with CT26 cells; six days after injection, the mice were given daily oral administration with MLN8237. Tumor volume was measured every three days. Two-way ANOVA is used for statistical analysis. Data are shown as mean ± SEM; *n* = 6. ****p-value < 0.0001. Mice were sacrificed, and tumor weight was measured on day 21 of post-subcutaneous injection. **C** The tumors were homogenized, and the tumor supernatants were then collected to analyze the secretion of IL-16 by ELISA. Student’s *t*-test is used for statistical analysis. Data are shown as mean ± SEM; *n* = 6. *p-value < 0.05. **D** A schematic illustration of MLN8237 and antibody treatment in the allogenic animal model is shown. CT26 was subcutaneously injected into 8-week-old Balb/c mice; 6 days after injection, the mice were orally administered MLN8237 daily, and IgG or anti-IL-16 antibodies were I.T. injected on day 8, day 11, and day 14 after subcutaneous injection. **E** Tumor volume was measured in control (blue), MLN8237 (orange), and MLN8237 + anti-IL-16 antibody (red) treated mice. **F**−**J** Mice were sacrificed, and tumor weight was measured on day 23 after subcutaneous injection. Tumor-infiltrating leukocytes (**F**), T cells (**G**), CD8^+^ T cells (**H**), CD4^+^ T cells (**I**), and PD1^+^ CD8^+^ T cells (**J**) were analyzed by flow cytometry. One-way ANOVA is used for statistical analysis. Data are shown as mean ± SEM; *n* = 3. *p-value < 0.05; **p-value < 0.01.

A previous report indicated that systemic inhibition of Aurora-A by specific inhibitors can promote antitumor immunity by directly inhibiting immunosuppressive cells in immunocompetent mice (ref. [39]). This result implies that Aurora-A may play different roles in regulating antitumor immunity between tumor cells and immunosuppressive cells. To clarify the role of Aurora-A inhibition in antitumor immunity, we investigated the effect of MLN8237 on CT26-derived tumor growth in immunocompetent mice. Indeed, MLN8237 inhibited tumor growth in 8-week-old Balb/c mice (Figs. 7A, B) and led to an increased IL-16 expression in CT26-derived tumors (Fig. 7C).

To determine whether the increased IL-16 impairs the therapeutic effect of MLN8237 (Fig. 7A–C), 8-week-old mice were subcutaneously injected with CT-26 and then co-treated with MLN8237 and anti-IL-16 antibodies (Fig. 7D). The results demonstrated that the combination of anti-IL-16 antibodies with MLN8237 results in decreased tumor growth (Fig. 7E), augmented infiltration of CD8^+^ T cells, and reduced infiltration of CD4^+^ T cells (Fig. 7F–J). However, the combination of anti-IL-16 antibodies and MLN8237 did not affect the infiltration of PD1^+^/CD8^+^ T cells (Fig. 7J). These results suggest that anti-IL-16 antibodies can enhance the therapeutic effect of MLN8237 by boosting antitumor immunity.

Taken together, in immune hot tumors, our findings indicated that Aurora-A plays a diverse role in immunosuppressive cells and tumor cells. Although the effect of MLN8237 on CT26-derived tumor growth differs from that of Aurora-A knockdown (Figs. 2A, B and 7A, B), the combination treatment of MLN8237 with anti-IL-16 antibodies enhances its therapeutic efficacy (Fig. 7E). Our studies demonstrate that tumor-intrinsic Aurora-A promotes antitumor immunity by inhibiting IL-16 production in high immune cell-infiltrated tumors. In those hot tumors, targeting Aurora-A can suppress cancer cell growth but attenuate antitumor immunity by upregulating IL-16 expression. The combination of Aurora-A inhibitors and anti-IL-16 antibodies may serve as an effective strategy for cancer therapy in the future.

## Discussion

Several reports have suggested that Aurora-A plays an oncogenic role in different cancers by promoting proliferation, migration, and invasion. Therefore, targeting Aurora-A is regarded as a potential strategy for cancer treatment. However, some studies have demonstrated that the inhibition of oncoproteins might reduce antitumor immunity in various cancers (ref. [17, 18]). Investigating the impact of targeting oncoproteins on immune cell functions in the TME has become crucial for cancer therapy assessment. This study found that tumor-intrinsic Aurora-A plays a diverse role in tumor progression. Our results revealed that lower lymphocyte infiltration in high tumor-intrinsic Aurora-A expressed CRC was associated with a poor prognosis; conversely, higher lymphocyte infiltration in high tumor-intrinsic Aurora-A expressed CRC was associated with a better prognosis. We demonstrated that tumor-intrinsic Aurora-A might promote antitumor immunity by inhibiting IL-16 secretion in immune hot TMEs (Supplementary Fig. S10). Decreased expression of Aurora-A can promote tumor growth by inhibiting the infiltration and cytotoxicity of CD8^+^ T cells. Therefore, neutralizing IL-16 with specific antibodies decreases tumor growth and enhances CD8^+^ T cell cytotoxicity in Aurora-A knockdown CT26-derived tumors.

Since only a few studies have reported on the role of tumor-intrinsic Aurora-A in antitumor immunity (ref. [40, 41]); in this study, by bioinformatic analysis (Supplementary Table S2), we hypothesized that tumor-intrinsic Aurora-A promotes cell growth and antitumor immunity by inhibiting immunosuppressive signaling pathways in tumors with high lymphocyte infiltration. This hypothesis was confirmed by allogenic animal models using immunodeficient and immunocompetent mice. Knockdown of tumor-intrinsic Aurora-A only promotes tumor growth in immune hot tumors but no significant effect is observed in immune cold tumors. Aurora-A in tumor cells can influence tumor growth by regulating antitumor immunity through inhibiting IL-16 expression in the immune hot TME.

A previous study demonstrated that tumor-intrinsic Aurora-A inhibits the cytotoxicity of CD8^+^ T cells by promoting PD-L1 expression in a triple-negative breast cancer (TNBC) model (ref. [41]). However, our results showed that tumor-intrinsic Aurora-A did not influence antitumor immunity in 4T1-derived tumors, which are known as immune cold tumors (ref. [33]). Another study demonstrated that inhibiting Aurora-A with systemic MLN8237 can promote CD8^+^ T cell infiltration by inducing IL-10 production (ref. [40]). However, the role of IL-10 in regulating anti-tumor immunity remains a subject of debate. The previous study indicated that the blockade of IL-10 can promote T cell-mediated cell death in human colorectal cancer liver metastases (ref. [42]). Our studies found that the knockdown of Aurora-A promotes the secretion of immunosuppressive molecules from CRC cells, such as CCL-2 and IL-6 (Supplementary Fig. S7). Although our results showed a slight increase in IL-10, the fold change of IL-10 expression was lower compared to the other immunosuppressive molecules; in our results, the treatment of MLN8237 inhibited the growth of CT26-derived tumors in 8-week-old mice (Fig. 7A, B). This suggests that the role of IL-10 in the Aurora-A knockdown CT26-derived tumor model is subtle.

A previous report has demonstrated that systemic inhibition of Aurora-A using MLN8237 (alisertib) inhibits the growth of 4T1-derived tumors via directly inhibiting immunosuppressive cells in immunocompetent mice (ref. [39]), suggesting that Aurora-A may play varied roles in different cell populations within the TME. This study reveals that tumor intrinsic Aurora-A can modulate anti-tumor immunity by negatively regulating IL-16 expression in the tumor microenvironment (TME) (Supplementary Fig. S10). Aurora-A knockdown impaired anti-tumor immunity in immunocompetent mice but did not affect tumor growth in immunodeficient mice. Systemic inhibition of Aurora-A by MLN8237 can suppress tumor growth in immunocompetent mice, with a concomitant increase of IL-16 in tumors (Fig. 7A–C). We hypothesized that systemic Aurora-A inhibition may impair MDSC function, thereby promoting anti-tumor immunity; the increased expression of IL-16 in systemic Aurora-A inhibitor-treated mice might counteract this beneficial effect. To explore this hypothesis in CRC patients with high Aurora-A expression and immune infiltration, combined treatment was conducted using MLN8237 and IL-16 neutralizing antibodies. The results suggested that combinational treatment can enhance the therapeutic efficacy of Aurora-A inhibition (Fig. 7E).

IL-16 has been identified as a chemoattractant for CD4^+^ T cells (ref. [43, 44]). Several studies have indicated that polymorphisms of IL-16 are correlated with chronic inflammatory diseases and cancers (ref. [44, 45]). In addition, some reports have shown that IL-16 is overexpressed in several cancers (ref. [46–48]). However, the roles of IL-16 in tumor progression are poorly understood, and the role of IL-16 in antitumor immunity within the TME remains unclear. Furthermore, whether anti-IL-16 antibodies can become effective cancer therapy drugs needs further investigation. This study found that neutralizing IL-16 in Aurora-A knockdown CT26-derived tumors can inhibit tumor growth by promoting the cytotoxicity of CD8^+^ T cells. Interestingly, IL-16 neutralization did not promote CD8^+^ T cell infiltration in Aurora-A knockdown CT26-derived tumors (Fig. 6E). This result implies that Aurora-A may regulate the infiltration of CD8^+^ T cells via other factors. A recent study indicated that inhibition of Aurora-A kinase impairs its therapeutic efficacy by impairing anti-tumor immunity, notably through the enhancement of PD-L1 expression (ref. [49]). Our unpublished data also confirmed that inhibition of Aurora-A kinase with MLN8237 can promote PD-L1 expression in CT26 and MC38 cells. These results imply that inhibition of tumor-intrinsic Aurora-A may impair anti-tumor immunity by enhancing various immune-suppressing signaling pathways.

This study demonstrated that tumor-intrinsic Aurora-A in cancer cells could promote antitumor immunity by inhibiting IL-16 secretion in immune hot tumors. We postulate that combining Aurora-A kinase inhibitors with IL-16 antibodies will be a useful therapeutic strategy for cancer patients with high tumor-intrinsic Aurora-A expression and high lymphocyte infiltration.

## Materials and methods

### Cell culture

CT26 and MC38 murine colorectal adenocarcinoma cells were kindly provided by Dr. Kwang-Yu Chang (National Health Research Institutes). HCT116 human colorectal carcinoma cell was purchased from the Bioresource Collection and Research Center. 4T1 murine breast cancer cell was purchased from the American Type Culture Collection. CT26, HCT116, and 4T1 cells were grown in RPMI-1640 medium (31800-022, GIBCO) supplemented with 10% fetal bovine serum (VW-8510-186, Avantor) and 1% Penicillin-Streptomycin (30-02-Cl, CORNING). MC38 cell was grown in DMEM (12800-017, GIBCO) supplemented with 10% fetal bovine serum, 1% Penicillin-Streptomycin, 1% NEAA (11140-050, GIBCO), and 1% HEPES (15630-080, GIBCO). All cells were maintained at 37 °C and 5% CO_2_.

### Lentivirus-mediated shRNA infection

Lentivirus-mediated shRNAs or cDNAs were purchased from the National RNAi Core Facility, Taipei, Taiwan. Cells were infected with lentivirus-mediated shRNAs or cDNAs supplemented with 8 μg/ml polybrene in a culture medium. After 24 h, infected cells were selected with 2−8 μg/ml puromycin for 72 h. After selection, cells were sub-cultured for at least one passage for the following assays.

### Preparation of cell extracts, western blot analysis

The total cell lysate was extracted using RIPA lysis buffer (50 mM Tris-HCl/pH 8.0, 150 mM NaCl, 0.5% sodium deoxycholate, 1% Nonidet P-40, 0.1% SDS, one mM DTT, ten mM β-glycerol phosphate, and one mM EGTA) supplemented with protease inhibitor cocktail (P8340, Sigma). Anti-IL-16 (ab180792, Abcam), anti-phospho-Aurora-A (2914, Cell Signaling), anti-Aurora-A (A1231, Sigma), anti-cleavage caspase-3 (9661, Cell Signaling) and anti-α-tubulin (T6199, Sigma) antibodies were used for western blot analysis.

### Reverse transcription-quantitative real-time polymerase chain reaction (RT-qPCR)

Total RNA was extracted using Trisure (BIO38032, Bioline) according to the manufacturer’s instructions. One to two μg of total RNA was transcribed into cDNA by High-Capacity cDNA Reverse Transcription Kit (4368814, Thermo), and 10−100 ng of cDNA was used for qPCR by SYBR Green kit (1708880, BioRad). For the sequence information of primers, please check Supplementary Table S1.

### Caspase3/7 activity assay

The caspase 3/7 activity was determined using the ApoLive-Glo™ Multiplex Assay kit (G6411, Promega) according to the manufacturer’s instructions. Briefly, cells were treated with 100 nM and 200 nM MLN8237 for 24, 48, and 72 h, and then the Caspase3/7 activity was measured.

### Cytokine array

Aurora-A knockdown cells were cultured for 72 h, and culture media were harvested for the following analysis. The cytokines were measured using Proteome Profiler Mouse Cytokine Array Kit, Panel A (ARY006, R&D system) according to the manufacturer’s instructions. The expression of cytokines was quantified by BIO-LAB software.

### ELISA

CT26 cells were treated with MLN8237 for 48 h, and culture media were collected for analysis. The tumor supernatants were collected from Aurora-A knockdown-derived CT26 tumor on day 21 post-subcutaneous injection. The sera of CRC patients were collected to measure the expression level of IL-16. The secreted IL-16 was measured using Mouse IL-16 ELISA Kit (ab201282, Abcam) and Human IL-16 ELISA Kit (EH259RB, Invitrogen) according to the manufacturer’s instructions.

### Reporter assay

The promoter region of m*IL-16* (GRCm39, chromosome 7, 83384732-83385731) was subcloned into a pGL3 vector. The pGL3-m*IL-16*-promoter was transiently transfected into lenti-sh*GFP*-infected, lenti-shm*aurka*-1-infected, and lenti-shm*aurka*-2-infected CT26 cells. The transcriptional activity of the *mIL-16* promoter was measured using Dual-Luciferase® Reporter Assay System (E1910, Promega) according to the manufacturer’s instructions.

### Animal tumor model, MLN8237, and antibody treatment

C57BL/6 and BALB/c mice (8−12 week-old) and NOD-SCID mice (6−8 week-old) were obtained from the Laboratory Animal Center at National Cheng Kung University (Tainan, Taiwan). All animal operations were approved by the Animal Welfare Committee at National Cheng Kung University (IACUC NO.108302 and NO. 111051). 1.5 × 10^5^ CT26 and 4T1 cells, and 5 × 10^5^ MC38 cells, were subcutaneously injected into matched-strain mice. Mice were sacrificed on day 16−24 post-subcutaneous injection, and the solid tumors were then harvested for assays. Tumor volume was measured every three days and calculated using the following formula: tumor volume (mm^3^) = (length x width^2)/2. BALB/c mice were daily oral administration with 40 mg/kg MLN8237 (A10004, Adooq) from day six after subcutaneous injection. 5 mg/kg (IT) or 10 mg/kg (IP) of mouse IgG (400264, BioLegend) or anti-IL-16 (519108, BioLegend) antibodies were intratumorally or intraperitoneally injected on day 8, day 11, and day 14 post-subcutaneous injection. 10 mg/kg of rabbit IgG (BE0090, BioXcell) or anti-CD8 (BE0117, BioXcell) antibodies were intraperitoneally injected on day 6, day 11, and day 16 post-subcutaneous injection. The animal experiments were blinded.

### Immune population analysis

Tumor tissues were collected from day 20 to day 23 post-subcutaneous injection for analyzing the immune population by flow cytometry and IHC staining. Tumors were homogenized by gentleMACS™ Dissociator according to the manufacturer’s instructions. The dissociated cells were blockaded with anti-CD16/CD32 (553141, BD) antibodies for 20 min. Anti-CD45 (563891), anti-CD3 (551163), anti-CD4 (553051), and anti-CD8 (560182) antibodies were purchased from BD Biosciences for analyzing the leukocytes, T cells, CD4^+^ T cells, and CD8^+^ T cells.

For analyzing Th1 and cytotoxic CD8^+^ T cells, the dissociated cells were stimulated with 20 ng/ml PMA, 1.33 μM ionomycin, and BD GolgiStop™ Protein Transport Inhibitor (554724, BD) for 7 h; the stimulated cells were stained with anti-CD45 (563891, BD), anti-CD4 (553051, BD), anti-CD8 (560182, BD) and anti-IFNY (563376, BD) antibodies. The percentage of tumor-infiltrating T cells, CD4^+^ cells, and CD8^+^ cells were analyzed by IHC staining using anti-CD3 (ab5690, Abcam), anti-CD4 (550280, BD), and anti-CD8 (100701, Biolegend) antibodies, respectively, and quantified by TissueFAXS software.

### In vitro CD8^+^ T-cell activity assay

Splenocytes were collected from 8 to10-week-old BALB/c mice. CD4^+^ T cells were depleted using Dynabeads™ Mouse CD4 kit (11445D, Invitrogen), and CD8^+^ T cells were isolated using Dynabeads™ Untouched™ Mouse CD8 Cells Kit (11417D, Invitrogen). Purified CD8^+^ T cells were cultured in conditioned media collected from control or m*aurka* knockdown CT26 cells and activated by anti-CD3 (553057, BD Biosciences) and anti-CD28 (553294, BD Biosciences) antibodies. The cytotoxicity of CD8^+^ T cells from splenocytes was analyzed by measuring the percentage of CD107^+^/CD8^+^ T cells by flow cytometry. Anti-CD45 (563891), anti-CD3 (551163), anti-CD4 (550280), anti-CD8 (560182), and anti-CD107 (564347) antibodies used for flow cytometry were purchased from BD Biosciences.

### Patient specimens

Clinical colorectal cancer specimens were obtained from National Cheng Kung University Hospital following the principle of the Declaration of Helsinki. The experimental protocol was approved by the Institutional Review Board (IRB) of National Cheng Kung University Hospital, Tainan, Taiwan (A-ER-100-403; A-ER-106-011; A-ER-110-537). The expression pattern of Aurora-A in CRC was obtained from our previous report (ref. [50]). The percentage of tumor-infiltrating lymphocytes and tumor-infiltrating CD8^+^ cells were determined by H&E and IHC staining and analyzed by two independent pathologists.

### RNA-Seq analysis

Total RNA was extracted from Aurora-A knockdown CT26 and MC38 cells using TRIsure™. All preparation procedures for RNA samples were carried out according to Illumina’s official protocol. The library construction was established using Agilent’s SureSelect Strand-Specific RNA Library Preparation Kit. The sequence was determined by Illumina’s sequencing-by-synthesis (SBS) technology (Illumina, USA). Sequencing data (FASTQ reads) were generated using Welgene Biotech’s pipeline based on Illumina’s base-calling program bcl2fastq v2.20. All experiment of RNA-Seq analysis was performed by Welgene Biotech (Taipei, Taiwan).

### Bioinformatics analysis

TCGA Cancer data was downloaded from the FireBrowse website. The gene expression data were used for immune score analysis by ESTIMATE and xCell web tools, respectively. The survival rate was performed by the SurvExpress database. Gene Enrichment analysis was performed using MetaCore and GSEA softwares.

### Supplementary information

Supplementary Materials

Original data

Checklist_2023.11.25

## Data Availability

All data presented in this study are available within the main text and supplementary materials and can be obtained from the corresponding authors upon reasonable request. The next-generation sequencing data was deposited in the GEO database (GSE247046).
