# Supplementary material for: Neutralizing IL-16 enhances the efficacy of targeting Aurora-A therapy in colorectal cancer with high lymphocyte infiltration through restoring anti-tumor immunity
Source: Cell Death Dis. 2024 Jan 30;15(1):103. doi: 10.1038/s41419-023-06381-z (PMC10828506; doi:10.1038/s41419-023-06381-z)
Supplement: Supplementary file 1 — Supplementary Materials [file 41419_2023_6381_MOESM1_ESM.pdf]

Table S1. Primer sequences used in this study.

Species	Gene		Sequence
Human	***IL-16***	Forward	5'-AGTTCCCTTCAGTCTGGTCAG-3'
		Reverse	5'-AGCACCTTCCTCCTTGTGTAAG-3'
	AURKA	Forward	5'-AAATACAGTCCCACCTTCGGC-3'
		Reverse	5'-CGAATGACAGTAAGACAGGGC-3'
	ACTIN	Forward	5'-CTGGACTTCGAGCAAGAGATG-3'
		Reverse	5'-TGATGGAGTTGAAGGTAGTTTCG-3'
Mouse	***il-16***	Forward	5'-ACGCAGACTTCATCCTCCAC-3'
		Reverse	5'-TATAGTCCATCCGTGCCTGC-3'
	actin	Forward	5'-ACTGGGACGACATGGAGAAG-3'
		Reverse	5'-GGTACGACCAGAGGCATACAG-3'
	gapdh	Forward	5'-AAGGGCTCATGACCACAGTC-3'
		Reverse	5'-CAGGGATGATGTTCTGGGCA-3'

Table S2. A higher expression level of intrinsic Aurora-A is associated with a better prognosis in cancer patients with higher immune cell infiltration.

Cancer Type	Immune Score	High AURKA	Low AURKA	P-Value
Acute Myeloid Leukemia	2619	Better prognosis	Poor prognosis	***
Kidney Renal Clear Cell Carcinoma	1083	Poor prognosis	Better prognosis	*
Lung Adenocarcinoma	968.5	Poor prognosis	Better prognosis	***
Pancreatic Ductal Adenocarcinoma	622.3	Poor prognosis	Better prognosis	***
Stomach Adenocarcinoma	613.3	Better prognosis	Poor prognosis	n.s.
Skin Cutaneous Melanoma	496.8	Poor prognosis	Better prognosis	n.s.
Lung Squamous Cell Carcinoma	471.2	Better prognosis	Poor prognosis	**
Head and Neck Squamous Cell Carcinoma	450	Poor prognosis	Better prognosis	n.s.
Cervical Carcinoma	339.7	Better prognosis	Poor prognosis	n.s.
Glioblastoma Multiforme	295.4	Better prognosis	Poor prognosis	**
Breast Cancer	213.4	Better prognosis	Poor prognosis	***
Kidney Renal Papillary Cell Carcinoma	113.1	Poor prognosis	Better prognosis	***
Colorectal Adenocarcinoma	73.83	Better prognosis	Poor prognosis	**
Bladder Urothelial Carcinoma	73.45	Poor prognosis	Better prognosis	n.s.
Liver Hepatocellular Carcinoma	27.88	Poor prognosis	Better prognosis	**
Esophageal Carcinoma	3.509	Poor prognosis	Better prognosis	n.s.
Thyroid Papillary Carcinoma	-71.89	Better prognosis	Poor prognosis	n.s.
Ovarian Serous Cystadenocarcinoma	-163.5	Poor prognosis	Better prognosis	n.s.
Uterine Corpus Endometrial Carcinoma	-238	Poor prognosis	Better prognosis	**
Adrenocortical Carcinoma	-459	Poor prognosis	Better prognosis	***
Low Grade Glioma	-557.9	Poor prognosis	Better prognosis	***
Paraganglioma and Pheochromocy	-694.6	Poor prognosis	Better prognosis	n.s.
Prostate Adenocarcinoma	-702.6	Poor prognosis	Better prognosis	*
Uterine Carcinosarcoma	-803.3	Poor prognosis	Better prognosis	n.s.

Supplementary Figure S1

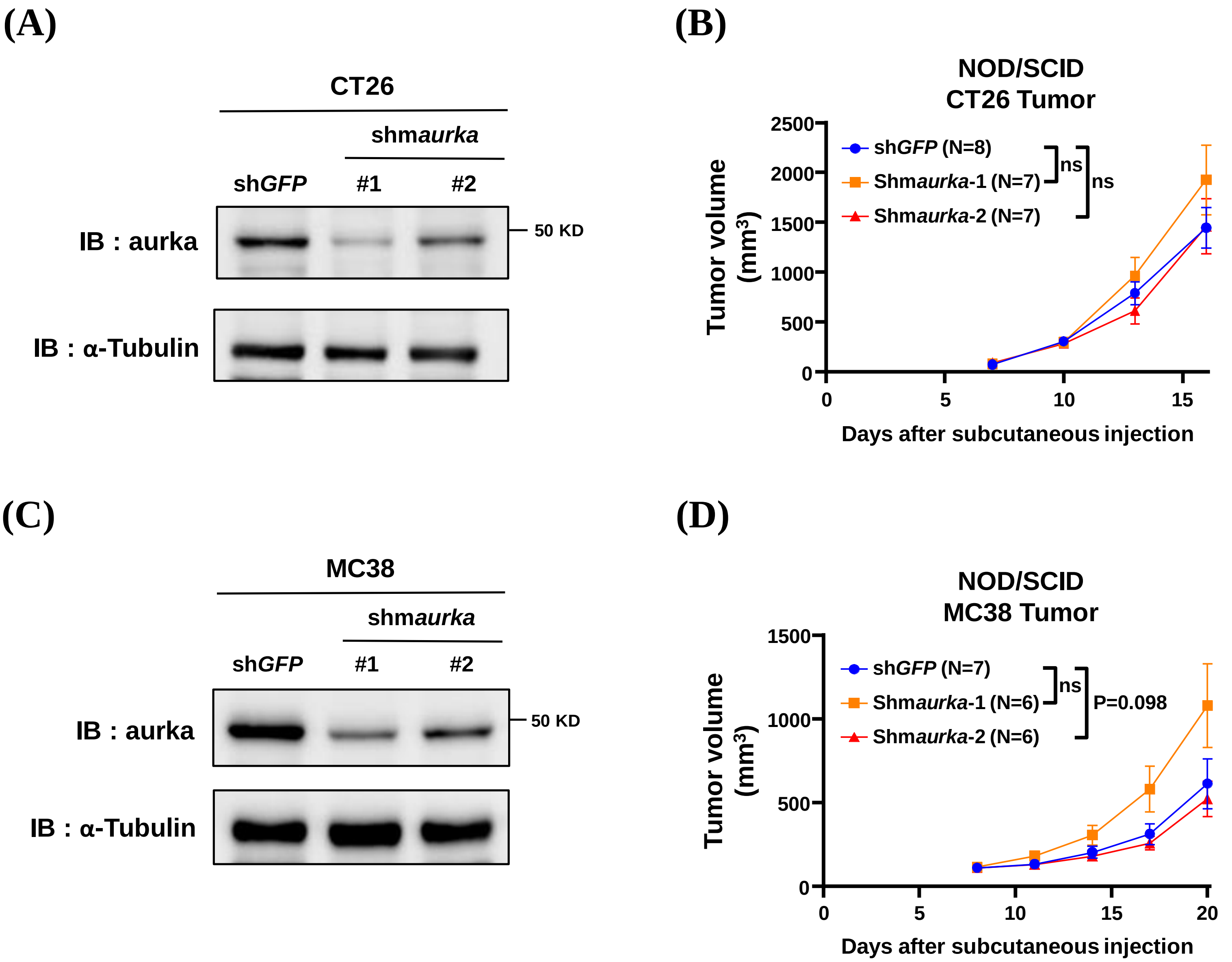

Supplementary Figure S2

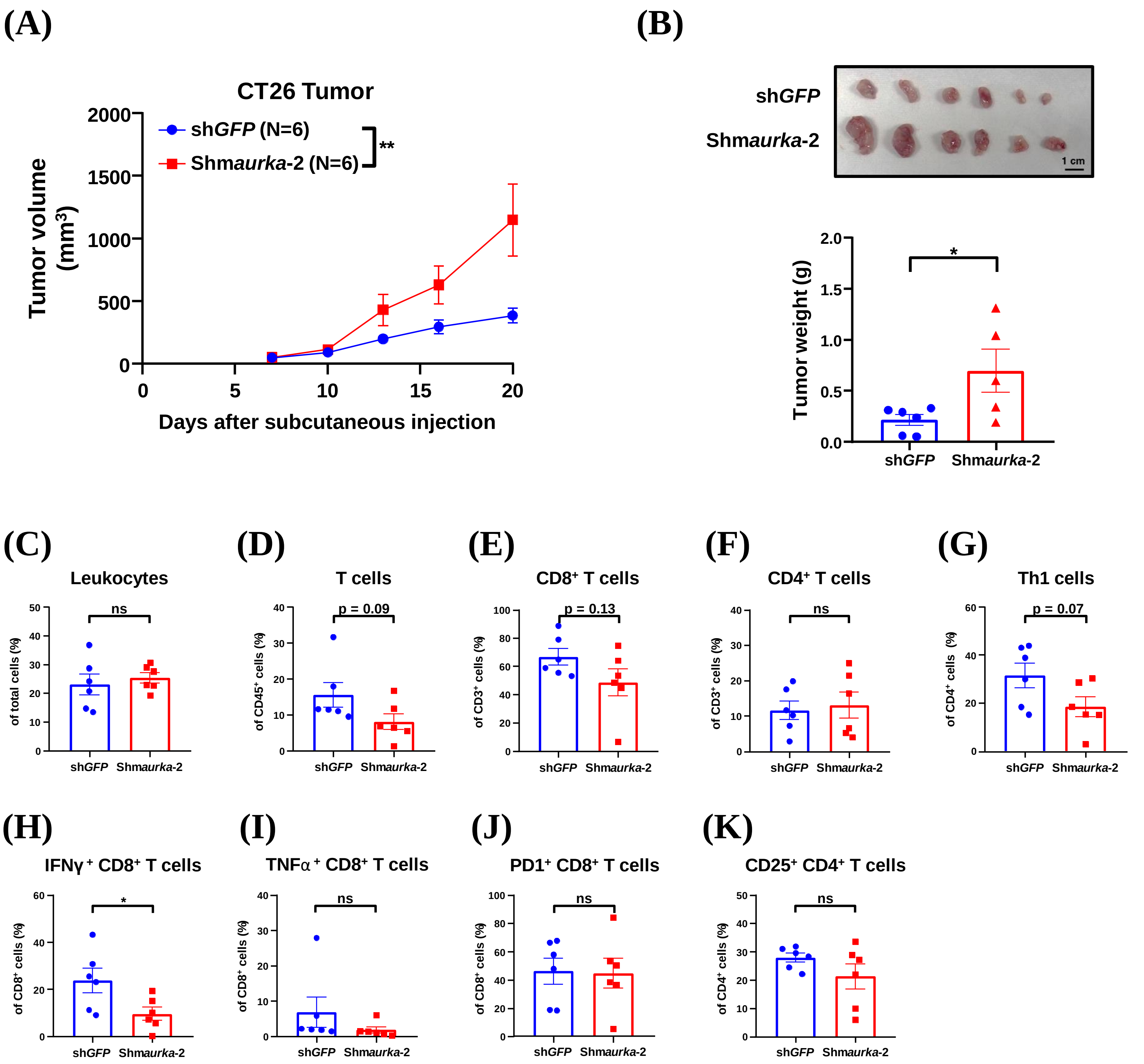

Supplementary Figure S3

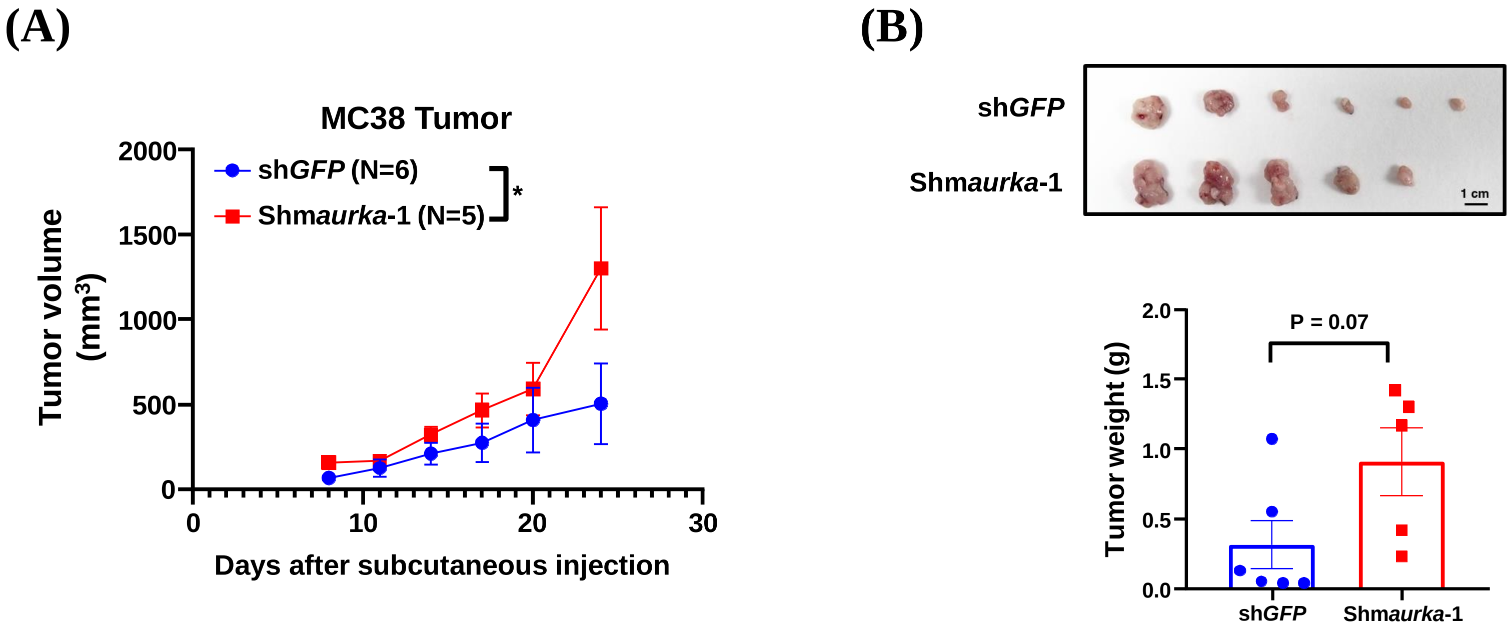

Supplementary Figure S4

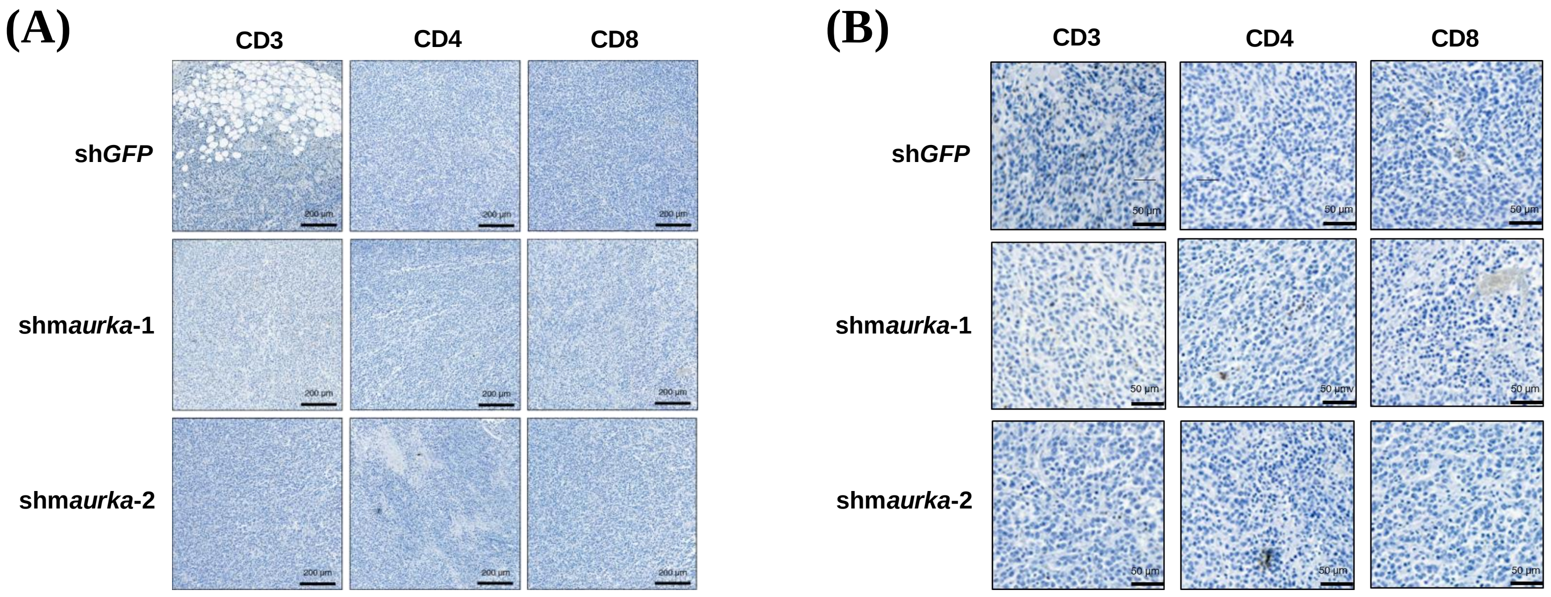

Supplementary Figure S5

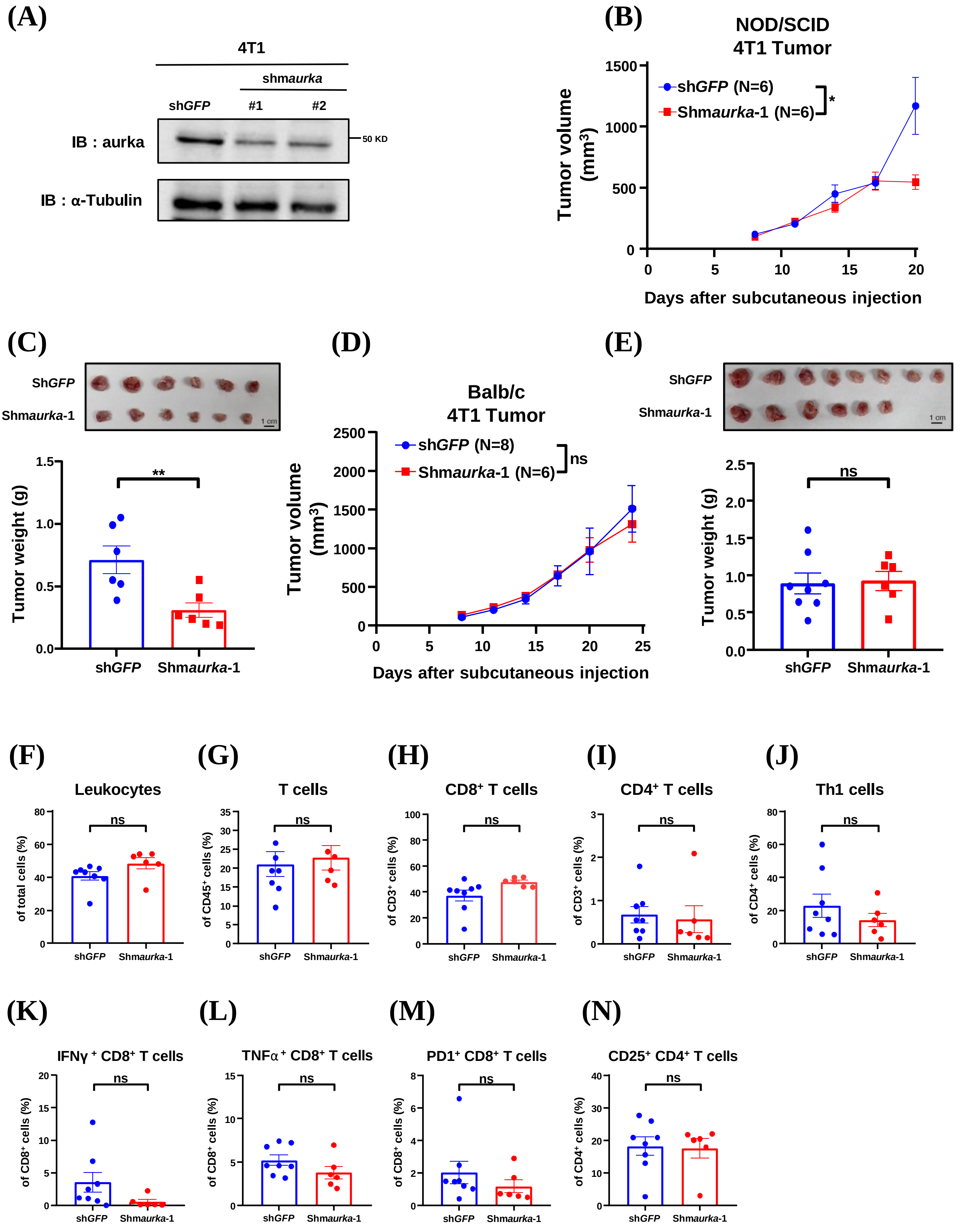

Supplementary Figure S6

(A)

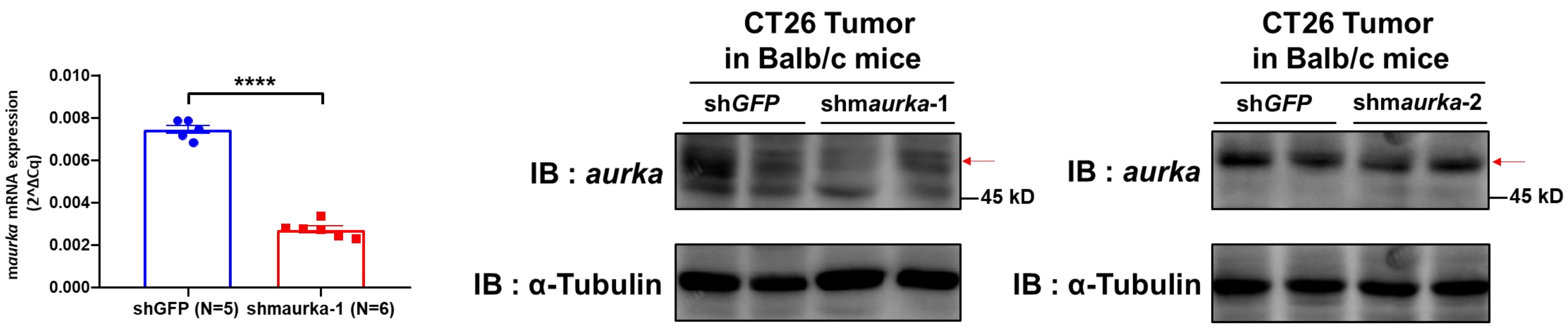

(B)

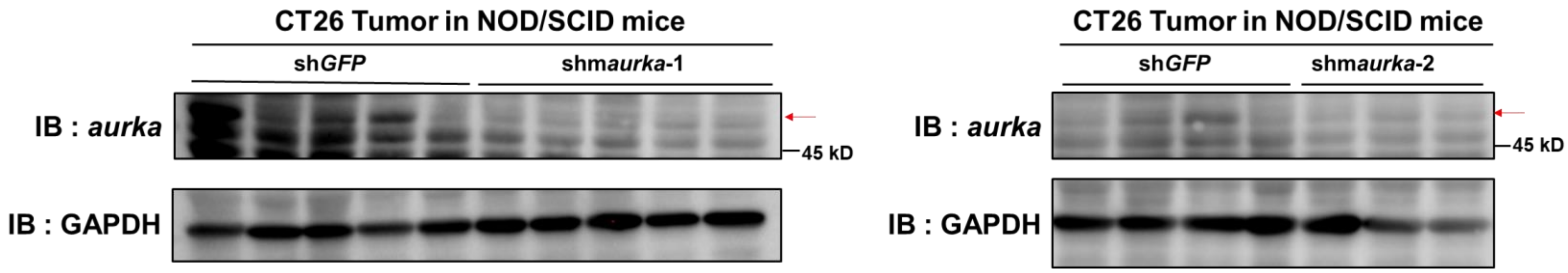

(C)

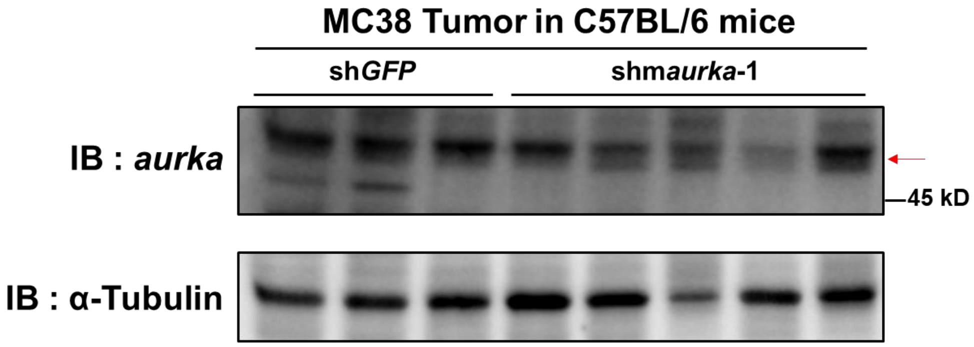

Supplementary Figure S6

(D)

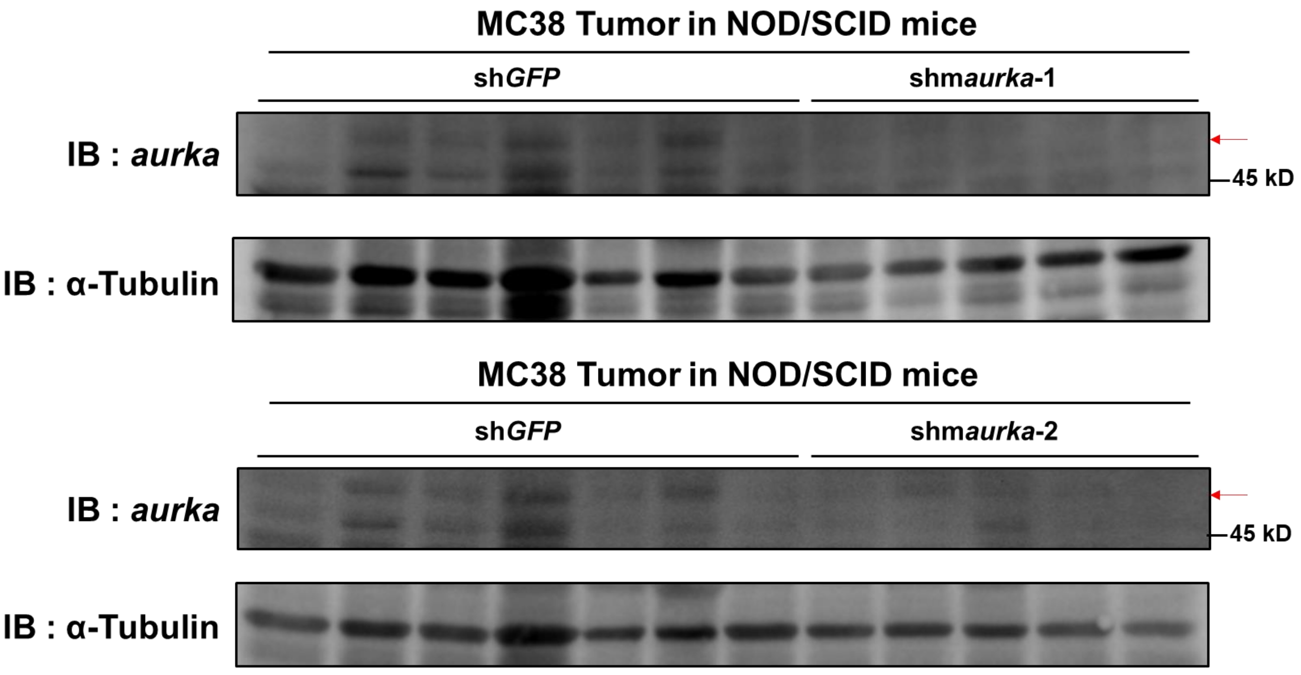

(E)

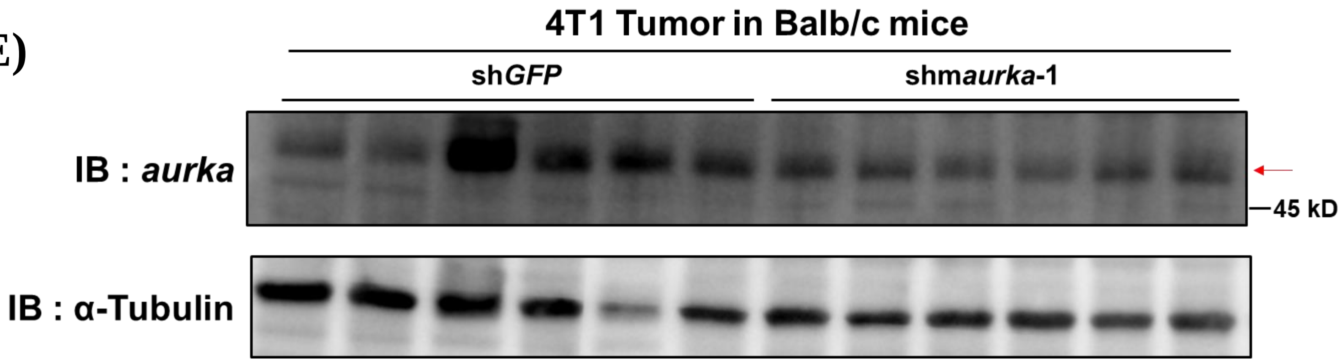

(F)

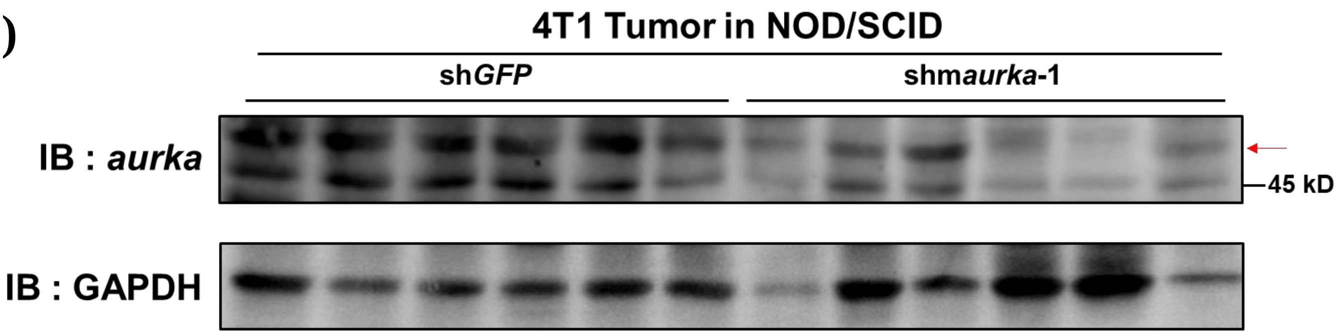

Supplementary Figure S7

(A)

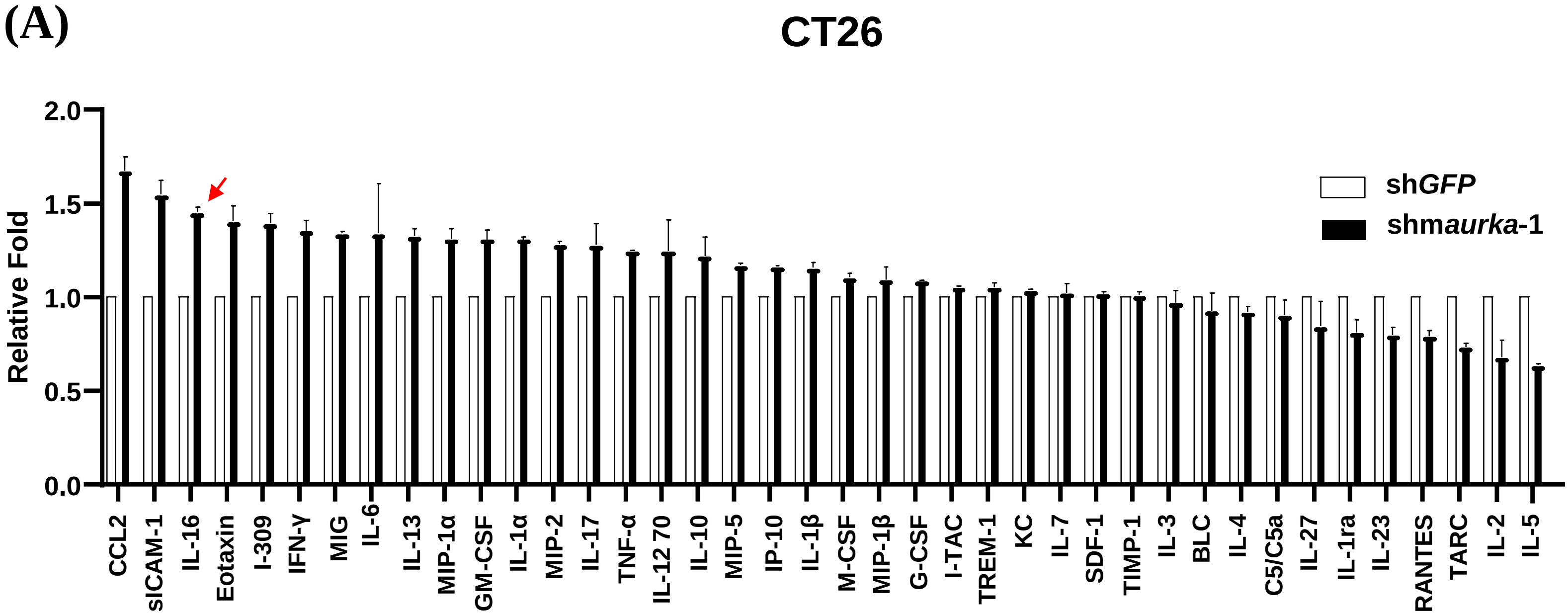

(B)

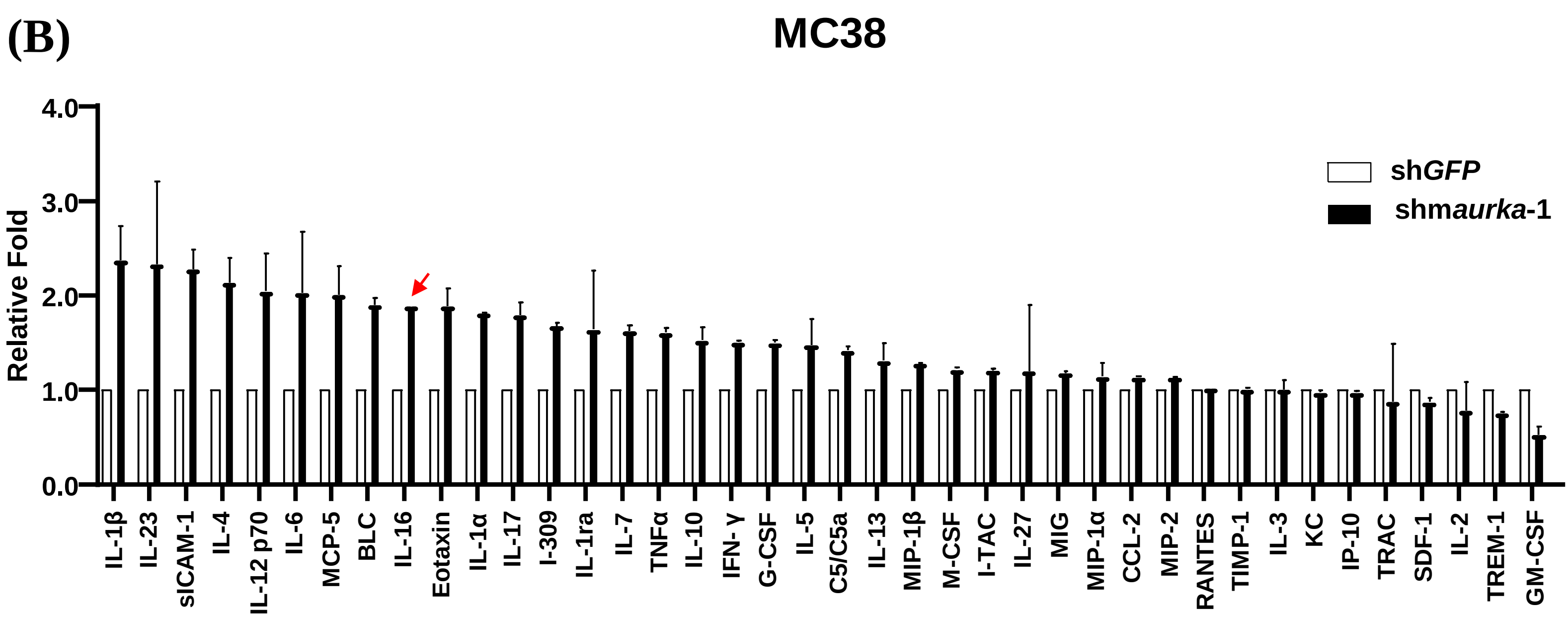

Supplementary Figure S8

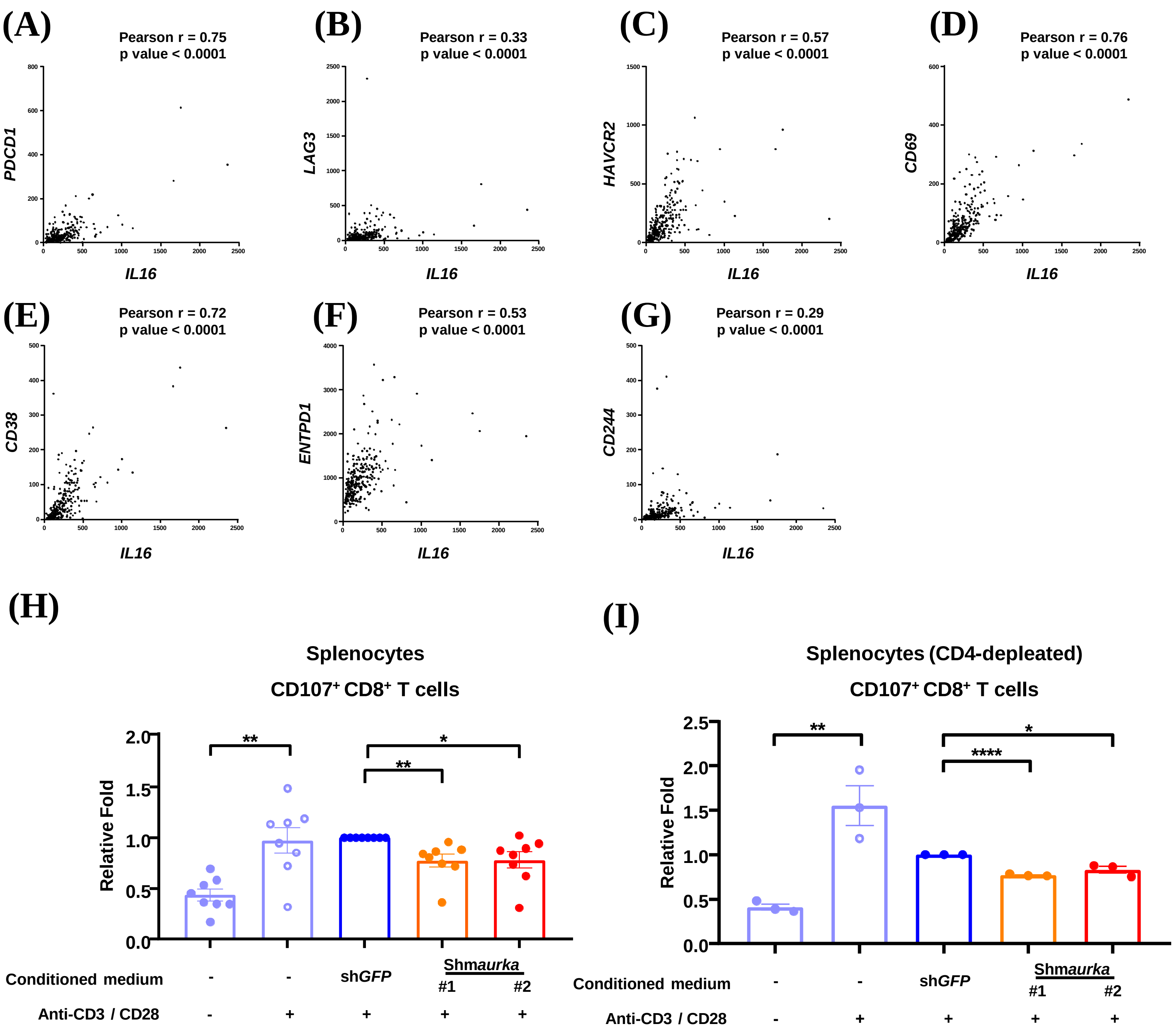

Supplementary Figure S9

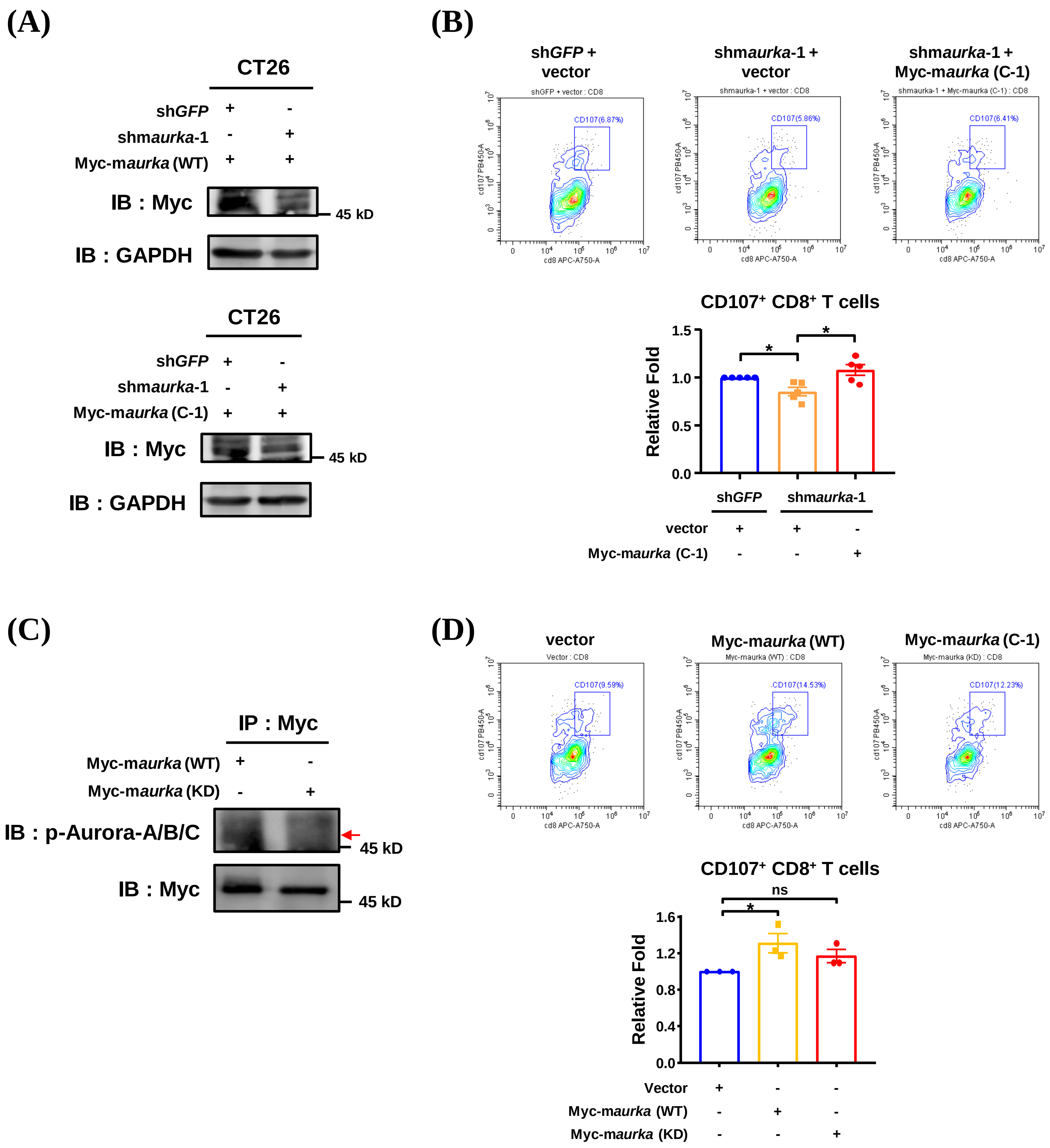

Supplementary Figure S10

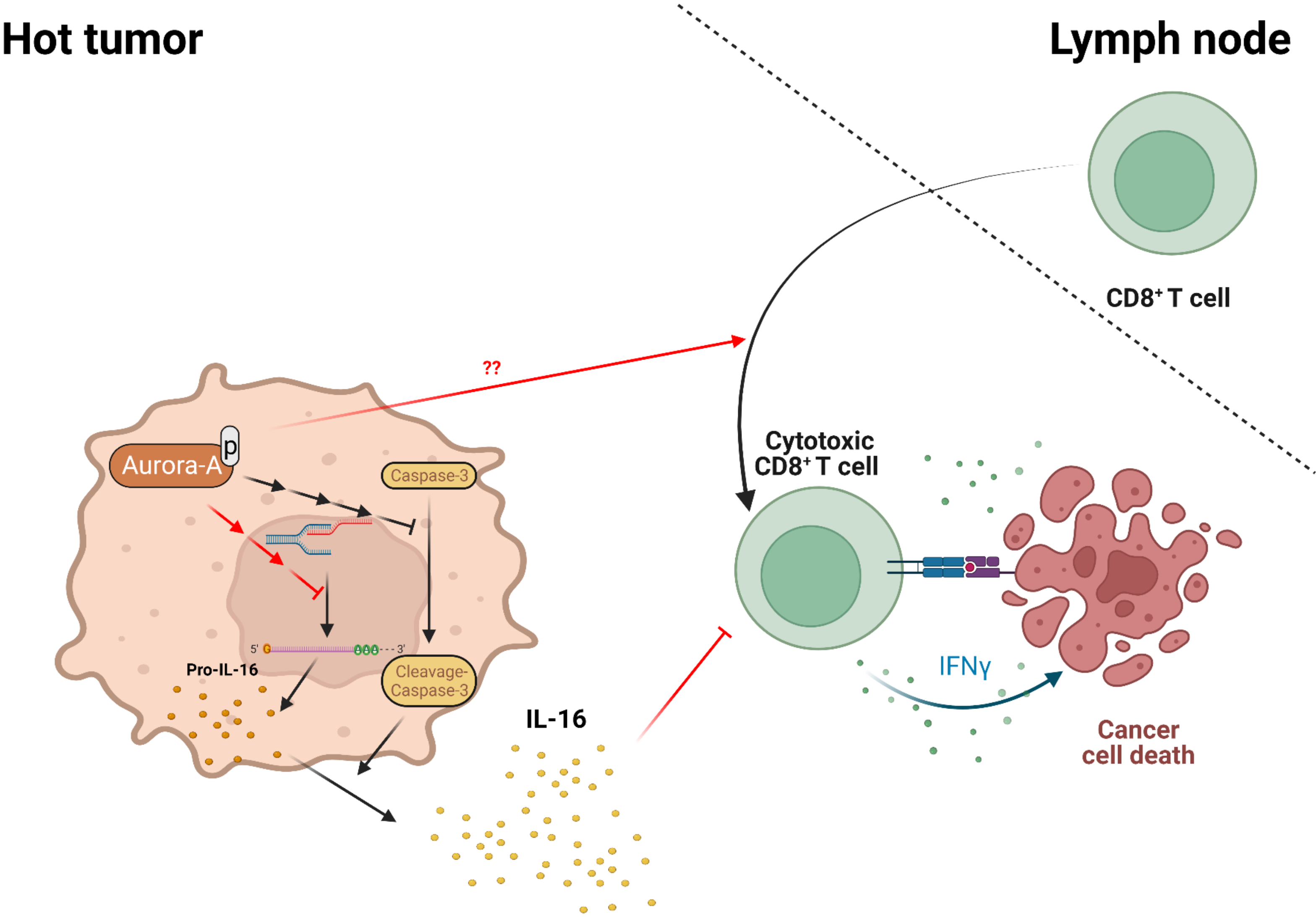

Supplementary Figure S1. Knockdown of Aurora-A in CT26 does not affect tumor growth in immunodeficient mice. In vivo tumor growth was evaluated by a subcutaneous allogenic animal model in NOD/SCID. **(A-B)** CT26 cells and **(C-D)** MC38 cells were infected with lenti-sh*GFP*, -shm*aurka*-1, and shm*aurka*-2. Cells were subcutaneously injected into NOD/SCID. Tumor volume was measured. Expression of mouse aurora-a (*aurka*) in CT26 (A) and MC38 cells (C) was evaluated by western blot. Two-way ANOVA is used for statistical analysis. Data are shown as mean ± SEM; n= 6~8 mice.

Supplementary Figure S2. Knockdown of Aurora-A in CT26 cells promotes tumor growth via inhibiting CD8$^+$ T cell activity in immunocompetent mice. CT26 cells infected with lenti-sh*GFP* and lenti-shm*aurka*-2 were subcutaneously injected into Balb/c mice. **(A)** Tumor volume was measured every three days. Two-way ANOVA is used for statistical analysis. Data are shown as mean ± SEM; n=6. **, p-value < 0.01. **(B)** Mice were sacrificed, and tumor weight was measured on day 20 of post-subcutaneous injection. Student's t-test is used for statistical analysis. Data are shown as mean ± SEM; n=6. *, p-value < 0.05. **(C)** Tumor-infiltrating leukocytes, **(D)** T cells, **(E)** CD8$^+$ T cells, **(F)** CD4$^+$ T cells, **(G)** Th1 cells (IFNγ$^+$ CD4$^+$ T cells), **(H)** IFNγ$^+$ CD8$^+$ T cells, **(I)** TNFα^+ CD8$^+$ T cells, **(J)** PD1$^+$ CD8$^+$ T cells, and **(K)** CD25$^+$ CD4$^+$ T cells were measured by flow cytometry. Student's *t*-test is used for statistical analysis. Data are shown as mean ± SEM; n=6. *, p-value < 0.05.

Supplementary Figure S3. Knockdown of Aurora-A in MC38 cells promotes tumor growth in immunocompetent mice. MC38 cells infected with lenti-sh*GFP* and lenti-shm*aurka*-1 were subcutaneously injected into C57BL/6 mice. **(A)** Tumor volume

was measured every three days. Two-way ANOVA is used for statistical analysis. Data are shown as mean ± SEM; n= 5 or 6. *, p-value < 0.05. **(B)** Mice were sacrificed, and tumor weight was measured on day 24 of post-subcutaneous injection. Student's t-test is used for statistical analysis. Data are shown as mean ± SEM; n= 5 or 6.

Supplementary Figure S4. Knockdown of Aurora-A in CT26 cells reduces the infiltration of CD8$^+$ T cells in immunocompetent mice. Tumor-infiltrating T cells (CD3), CD4$^+$ T cells, and CD8$^+$ T cells were analyzed by IHC staining. **(A)** scale bar 200 μm; **(B)** scale bar 50 μm.

Supplementary Figure S5. Knockdown of Aurora-A in 4T1 breast cancer cells cannot inhibit anti-tumor immunity in cold tumors. *In vivo* 4T1 tumor growth was evaluated by a subcutaneous allogenic animal model in NOD/SCID and Balb/c mice. 4T1 cells were infected with lenti-sh*GFP* and lenti-shm*aurka*-1. **(A)** The expression of mouse aurora-a (*aurka*) was evaluated by western blot analysis. **(B-E)** Tumor volume was measured every three days in NOD/SCID (B) and Balb/c mice (D). Two-way ANOVA is used for statistical analysis. Data are shown as mean ± SEM; n= 6-8 mice. *, p-value < 0.05. Tumor weight was measured on day 24 of post-subcutaneous injection (C, E). **(F)** Tumor-infiltrating leukocytes, **(G)** T cells, **(H)** CD8$^+$ T cells, **(I)** CD4$^+$ T cells, **(J)** Th1 cells (IFNγ$^+$ CD4$^+$ T cells), **(K)** IFNγ$^+$ CD8$^+$ T cells, **(L)** TNFa^+ CD8$^+$ T cells, **(M)** PD1$^+$ CD8$^+$ T cells, and **(N)** CD25$^+$ CD4$^+$ T cells were measured by flow cytometry. Data are shown as mean ± SEM; n= 6 or 8 mice. Student's t-test is used for statistical analysis.

Supplementary Figure S6. The expression level of Aurora-A in mouse tumor tissues. (A) CT26-bearing tumors harvested from Figure 2B (shm*aurka*-1) and Supplementary Figure S2B (shm*aurka*-2) were collected to evaluate the expression of Aurora-A by RT-qPCR and western blot analysis. **(B)** Expression of Aurora-A in CT26-bearing tumors harvested from Supplementary S1B was determined by western blot analysis. **(C-D)** Aurora-A expression in MC38-bearing tumors harvested from Supplementary S3B (C) and S1D (D) was evaluated by western blot analysis. **(E-F)** Aurora-A expression in 4T1-bearing tumors harvested from Supplementary S5C (E) and S5E (F) was evaluated by western blot analysis.

Supplementary Figure S7. The quantification result of cytokine array analyses of culture media from Aurora-A knockdown CT26 and MC38 cells. Lenti-sh*GFP*-infected and lenti-shm*aurka*-1-infected CT26 (A) and MC38 (B) cells were cultured for 72 h, and the cultured media were then collected for cytokine array analysis. The red arrows indicate the expression of IL-16.

Supplementary Figure S8. Aurora-A directly affects CD8^{+} T cell activity by down-regulating interleukin-16. (A-G) The correlation between *IL-16* and inhibitory checkpoint genes was determined by Pearson correlation analysis using the TCGA COAD dataset. The expression of inhibitory checkpoint genes was *PDCD1* (A), *LAG3* (B), *CD244* (C), *CD69* (D), *CD38* (E), *ENTPD1* (F), and *HAVCR2* (G). **(H)** Splenocytes were purified from Balb/c mice, and then cultured with conditioned media collected from CT26 cells infected with lenti-sh*GFP,* lenti-shm*aurka*-1, and lenti-shm*aurka*-2 for 72 h. Splenocytes were cultured in conditioned media for 48 h. **(I)** CD4^{+} T cells were depleted in the splenocytes and cultured in conditioned media for 48 h. The activity of

cytotoxic $CD8^+$ T cells ($CD107^+/CD8^+$) in splenocytes was measured by flow cytometry. The relative fold change of $CD107^+/CD8^+$ T cells is shown. Student's *t*-test is used for statistical analysis. Data are shown as mean ± SEM; n= 3 or 8 mice. *, p-value < 0.05; **, p-value < 0.01; ****, p-value < 0.0001.

Supplementary Figure S9. Tumor-intrinsic Aurora-A enhances $CD8^+$ T cell activity in a kinase-dependent manner. (A-B) shm*aurka*-1-resistant Myc-m*aurka* (C-1) construct was generated by mutating the targeting sequence. Western blot analysis was conducted to assess Myc-Aurora-A expression, confirming the resistance of Myc-m*aurka* (C-1) to shm*aurka*-1 (A). Splenocytes purified from BALB/c mice were co-cultured with CT26 cells infected with either lenti-sh*GFP* or lenti-shm*aurka*-1, and transiently transfected with vector or Myc-m*aurka* (C-1) (B). **(C-D)** Myc-m*aurka* (KD) was designed by mutating lysine 175 to methionine in a conserved region, corresponding to lysine 162 in human Aurora-A. CT26 cells were transiently transfected with either Myc-m*aurka* (WT) or Myc-m*aurka* (KD). Immunoprecipitation (IP)-western blot was conducted to assess the kinase activity of Myc-m*aurka* (WT) and Myc-m*aurka* (KD) (C). Splenocytes purified from BALB/c mice were co-cultured with CT26 cells transiently transfected with the vector, Myc-m*aurka* (WT), or Myc-m*aurka* (KD) (D). Anti-CD3/CD28 antibodies were used to activate T cells. The cytotoxicity of $CD8^+$ T cells in the splenocytes was measured by flow cytometry using anti-CD107a

antibodies. Data showing the relative fold change of $CD107^+/CD8^+$ T cells are presented. Statistical analysis was conducted using Student's t-test. Data are expressed as mean ± SEM; *, $p < 0.05$; n = 5.

Supplementary Figure S10. The model of Aurora-A-mediated anti-tumor immunity in CRC. In CRC tumors, overexpressed Aurora-A can reduce *IL-16* transcripts and attenuate mature IL-16 formation via inhibiting caspase-3 activity. Mature IL-16 can directly impair the cytotoxicity of $CD8^+$ T cells. Therefore, in CRC with higher lymphocyte infiltration, overexpressed Aurora-A promotes anti-tumor immunity by enhancing the activity of cytotoxic $CD8^+$ T cells. Figures were created by using BioRender (https://www.biorender.com/).
