# Supplementary material for: Neutralizing IL-16 enhances the efficacy of targeting Aurora-A therapy in colorectal cancer with high lymphocyte infiltration through restoring anti-tumor immunity
Source: Cell Death Dis. 2024 Jan 30;15(1):103. doi: 10.1038/s41419-023-06381-z (PMC10828506; doi:10.1038/s41419-023-06381-z)

Figure 4.

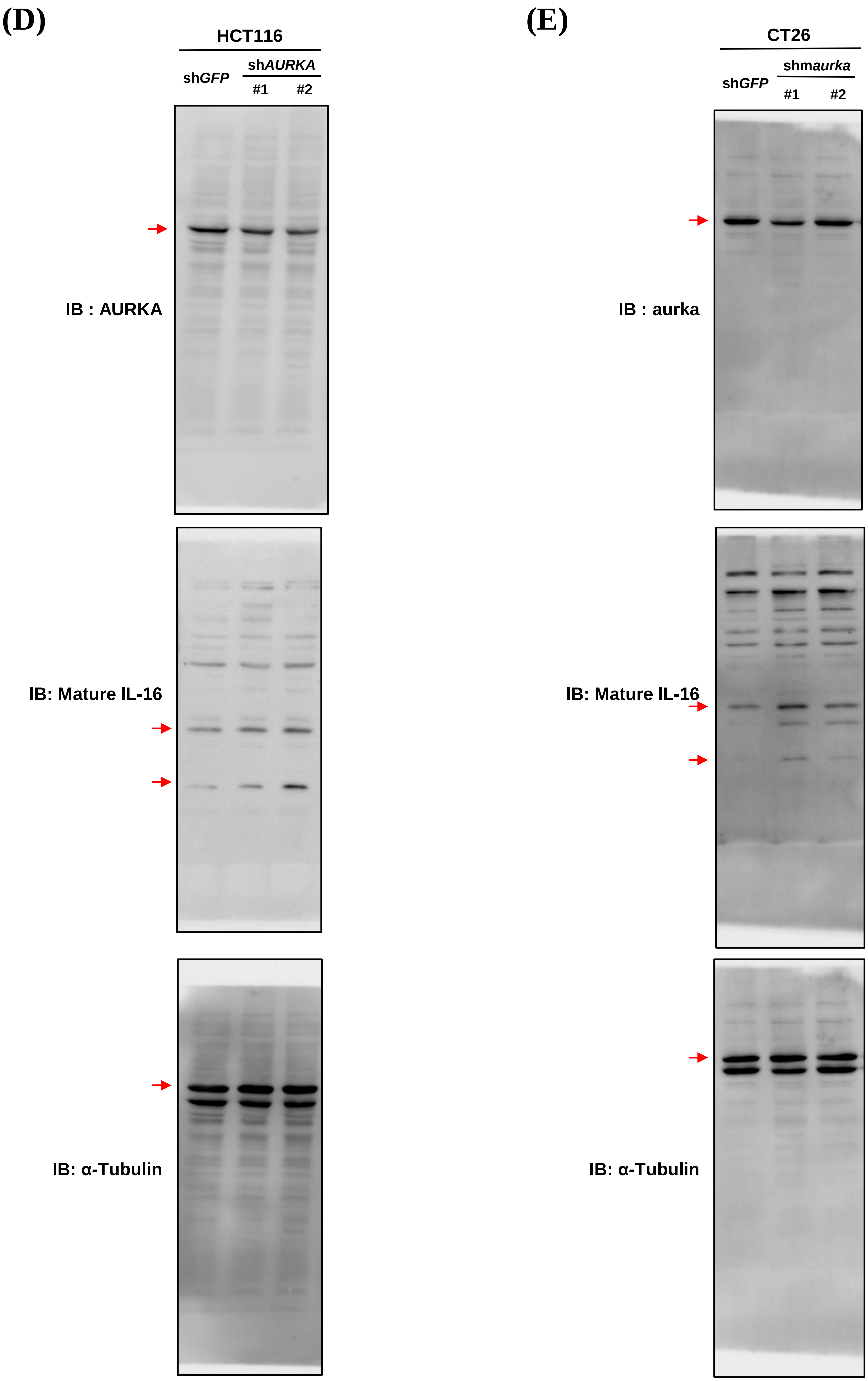
(D)
HCT116
shGFP
shAURKA
#1
#2
IB : AURKA
IB: Mature IL-16
IB: α-Tubulin
(E)
CT26
shGFP
shmaurka
#1
#2
IB : aurka
IB: Mature IL-16
IB: α-Tubulin

Figure 5.

(A)

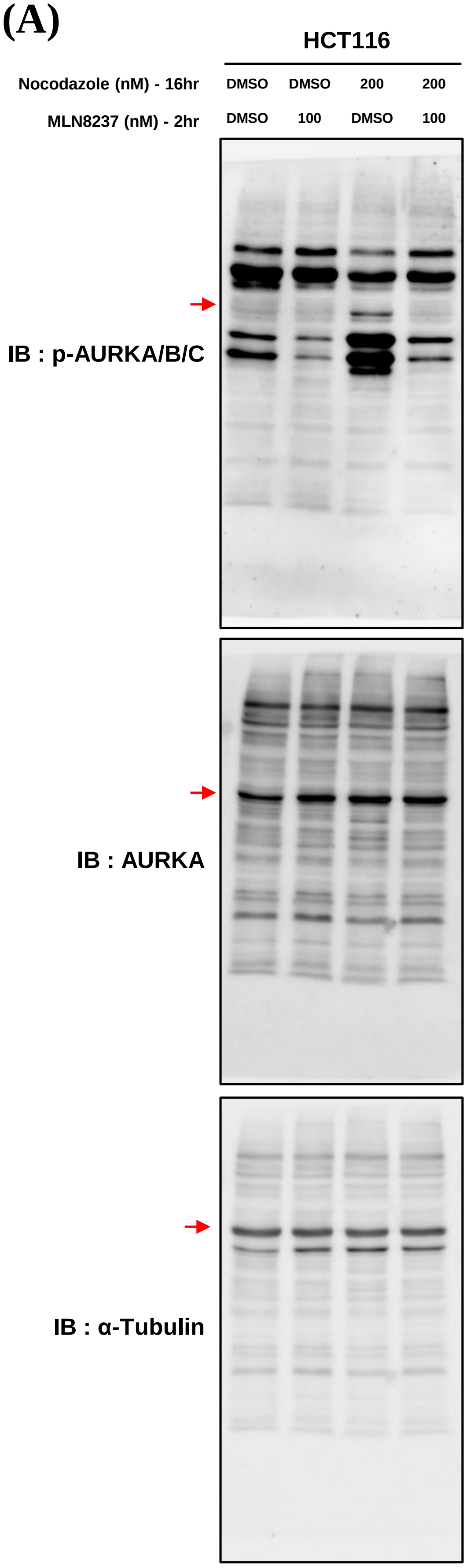
HCT116
Nocodazole (nM) - 16hr
DMSO
DMSO
200
200
MLN8237 (nM) - 2hr
DMSO
100
DMSO
100
IB : p-AURKA/B/C
IB : AURKA
IB : α-Tubulin

(E)

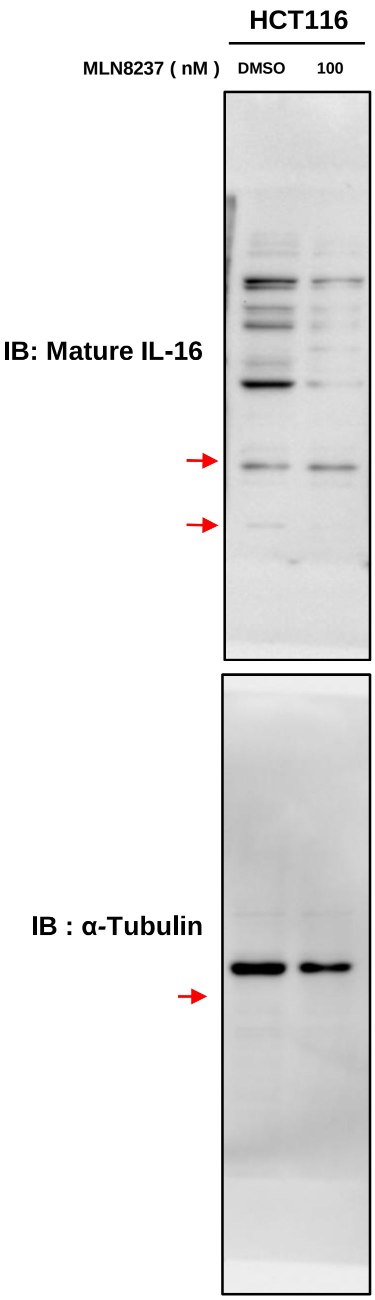
HCT116
MLN8237 (nM)
DMSO
100
IB: Mature IL-16
IB : α-Tubulin

Figure 5.

(F) (G)

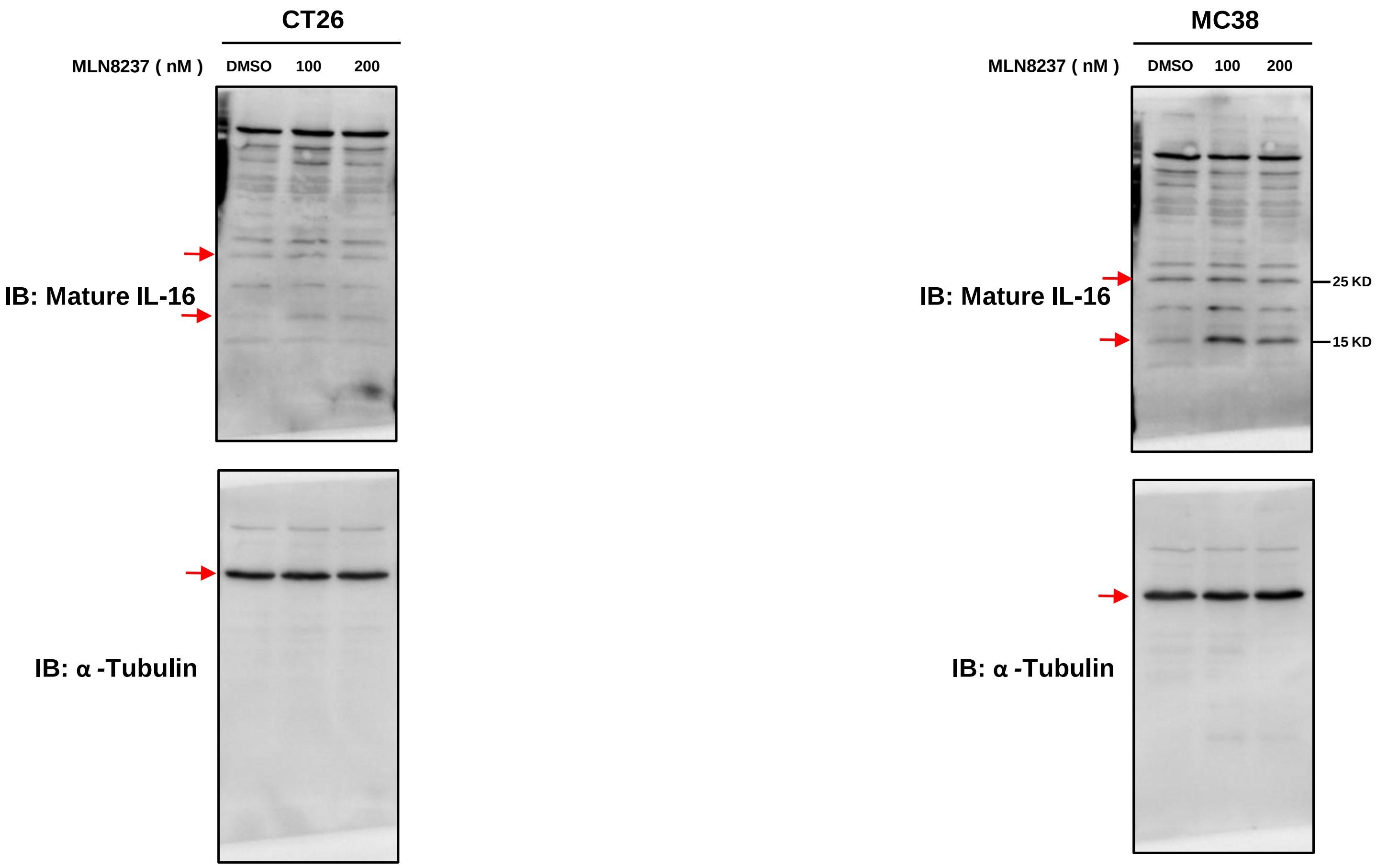
CT26
MLN8237 (nM)
DMSO
100
200
IB: Mature IL-16
IB: α -Tubulin
MC38
MLN8237 (nM)
DMSO
100
200
IB: Mature IL-16
25 KD
15 KD
IB: α -Tubulin

Figure 5.

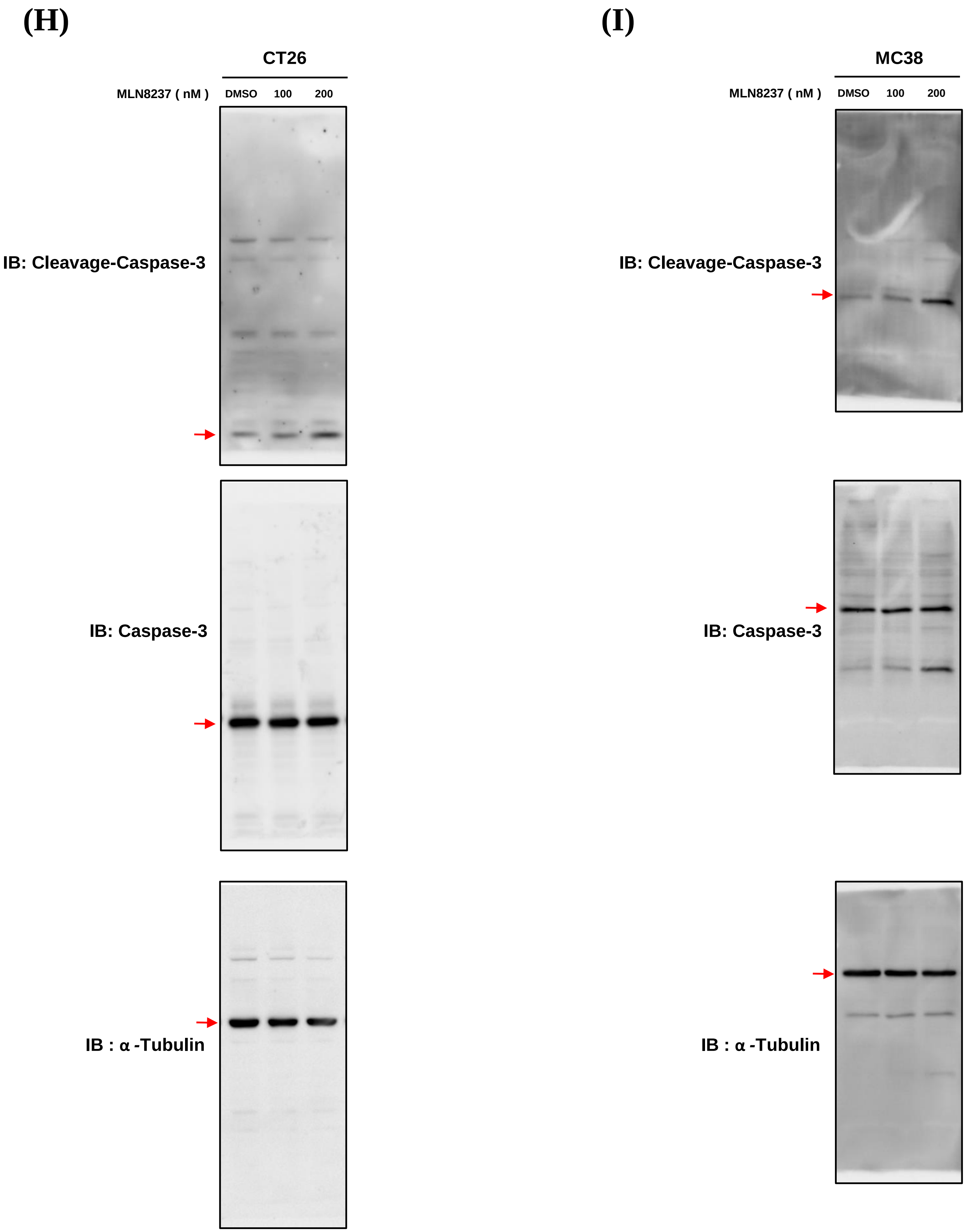
(H)
CT26
MLN8237 (nM)
DMSO
100
200
IB: Cleavage-Caspase-3
IB: Caspase-3
IB : α -Tubulin
(I)
MC38
MLN8237 (nM)
DMSO
100
200
IB: Cleavage-Caspase-3
IB: Caspase-3
IB : α -Tubulin

Supplementary Figure S1.

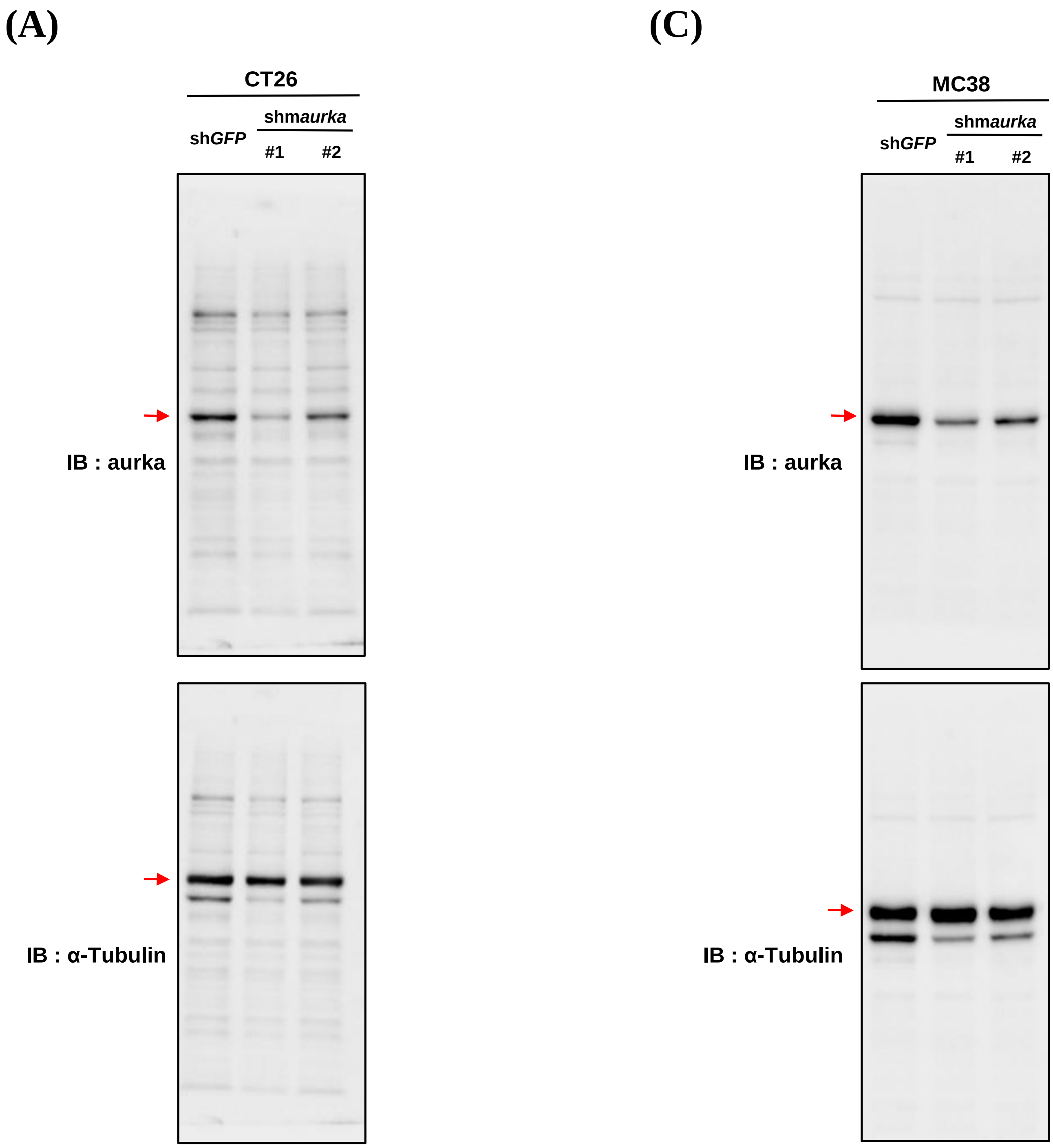

Supplementary Figure S5.

(A)

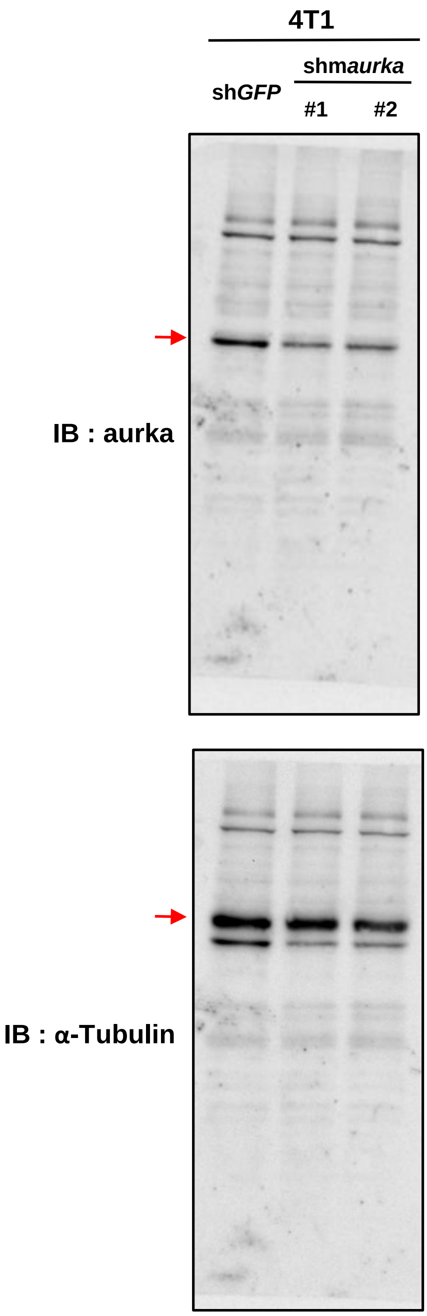

Supplementary Figure S6.

(A)

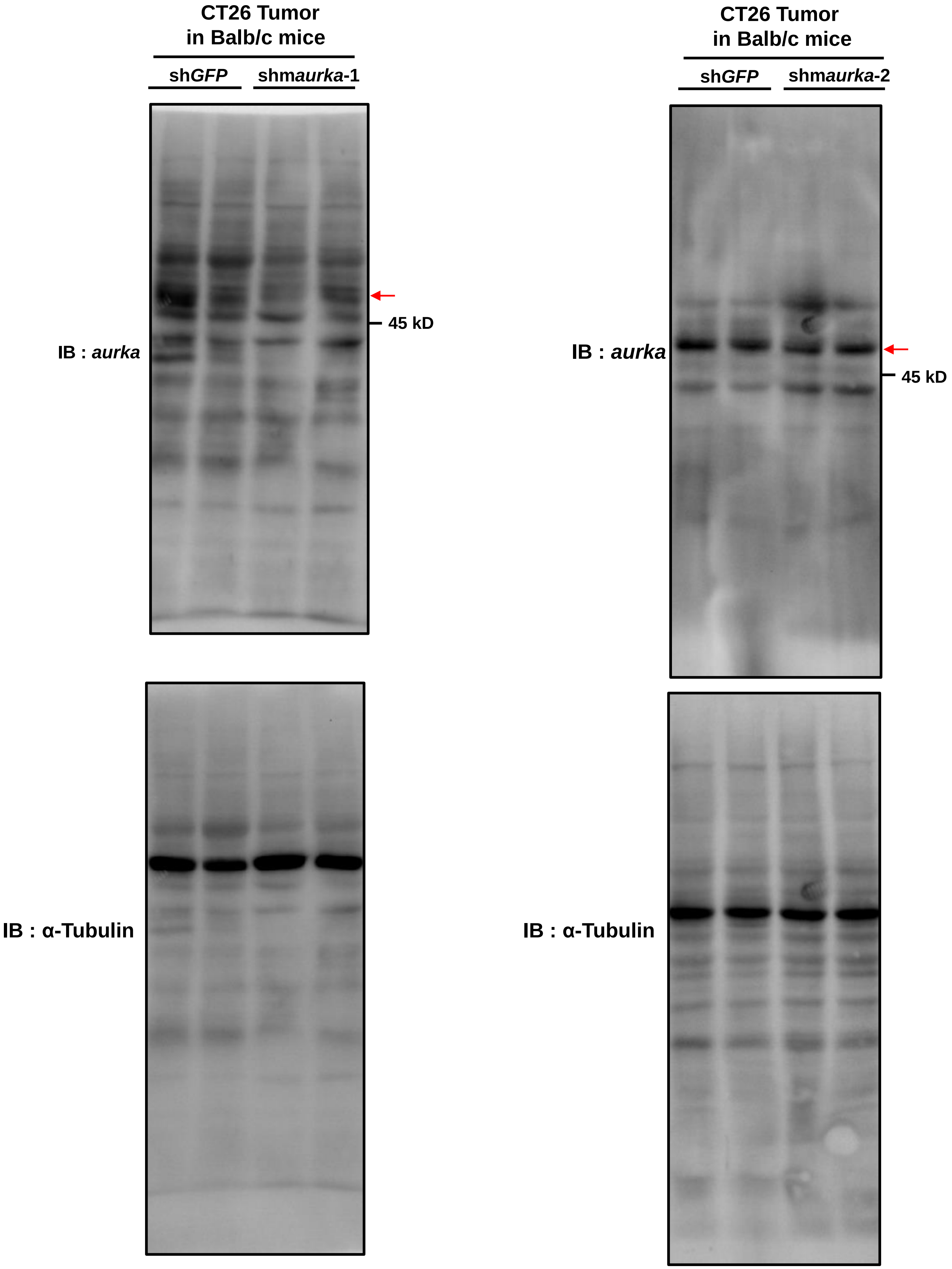

Supplementary Figure S6.

(B)

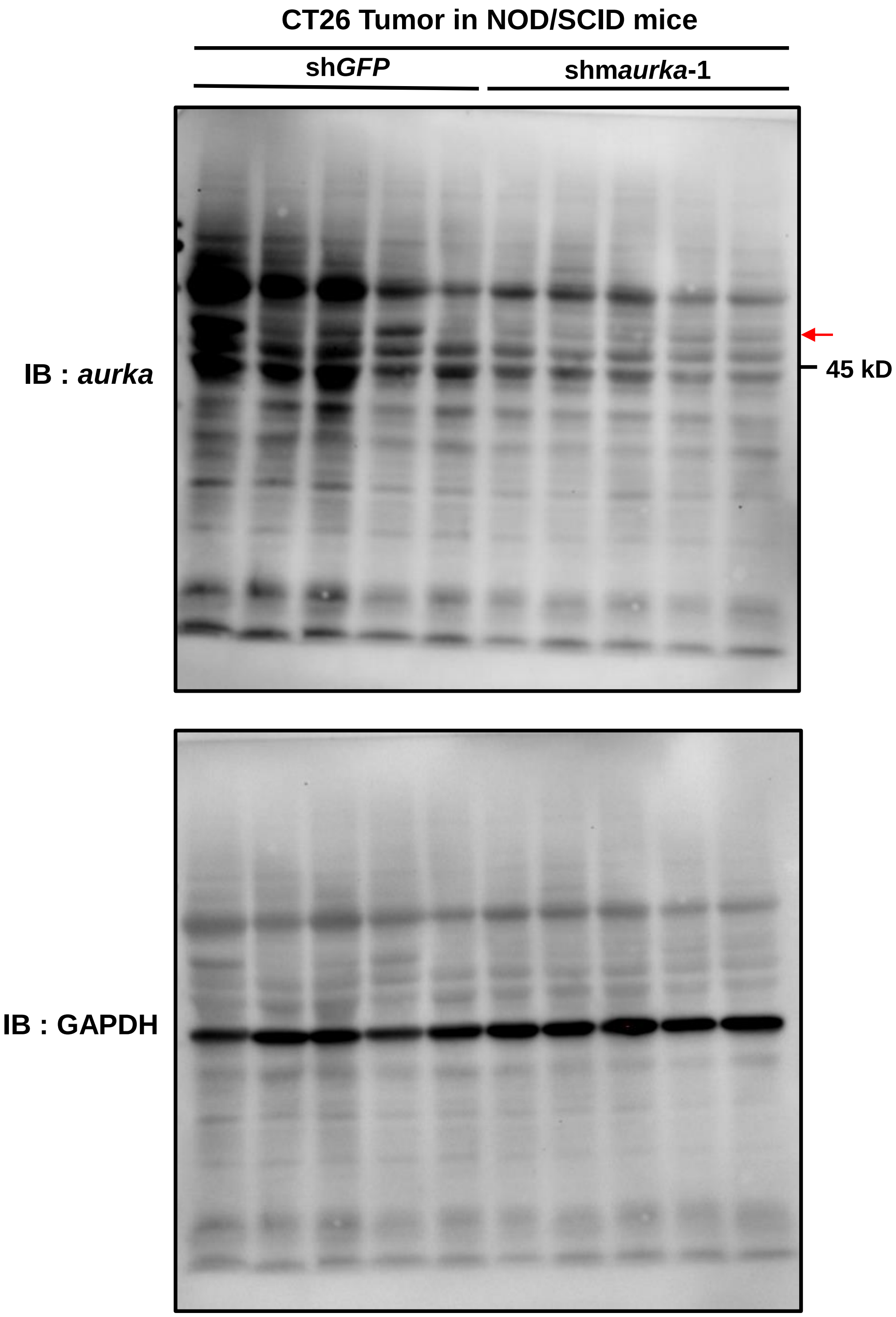

Supplementary Figure S6.

(B)

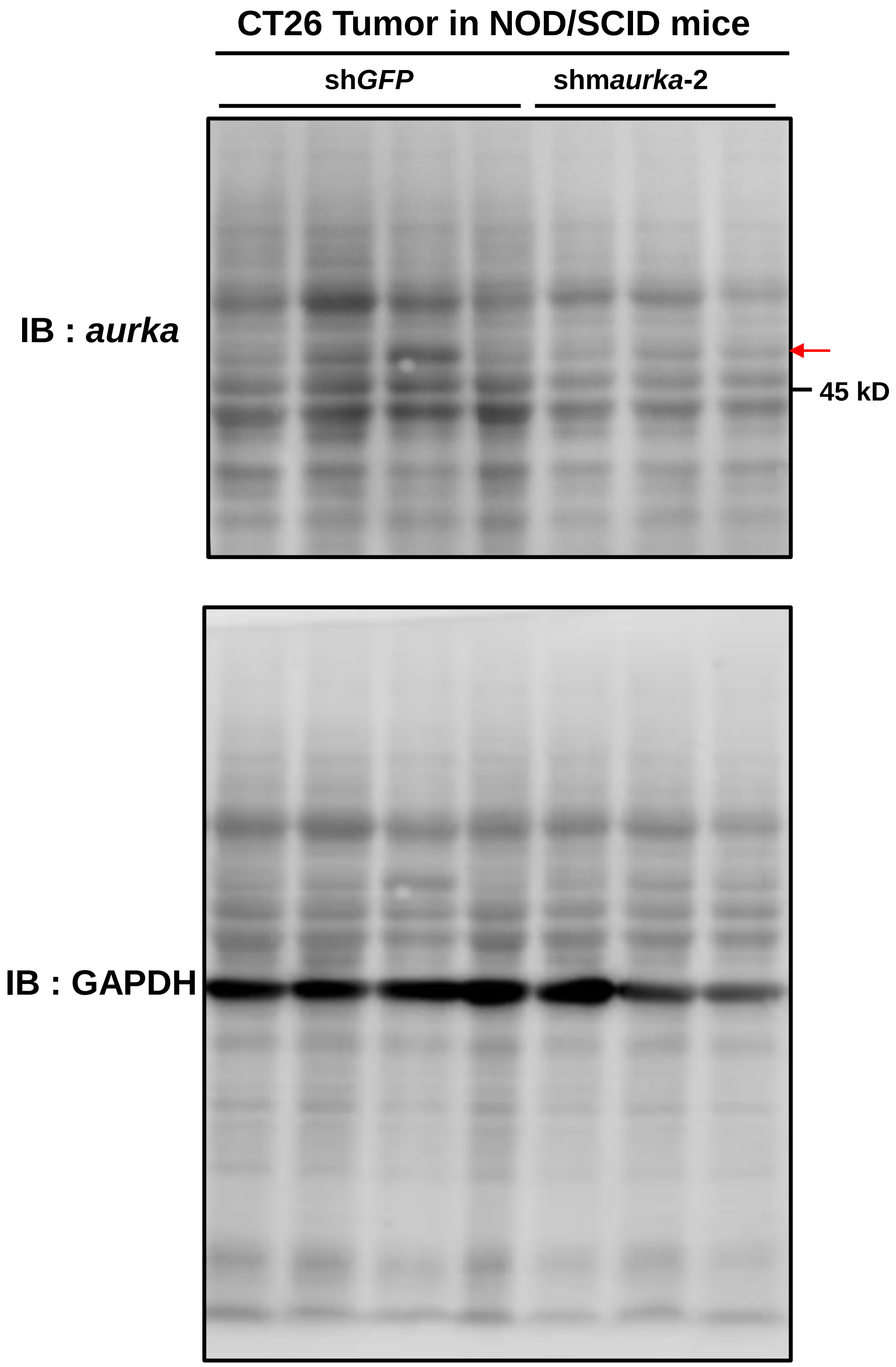

Supplementary Figure S6.

(C)

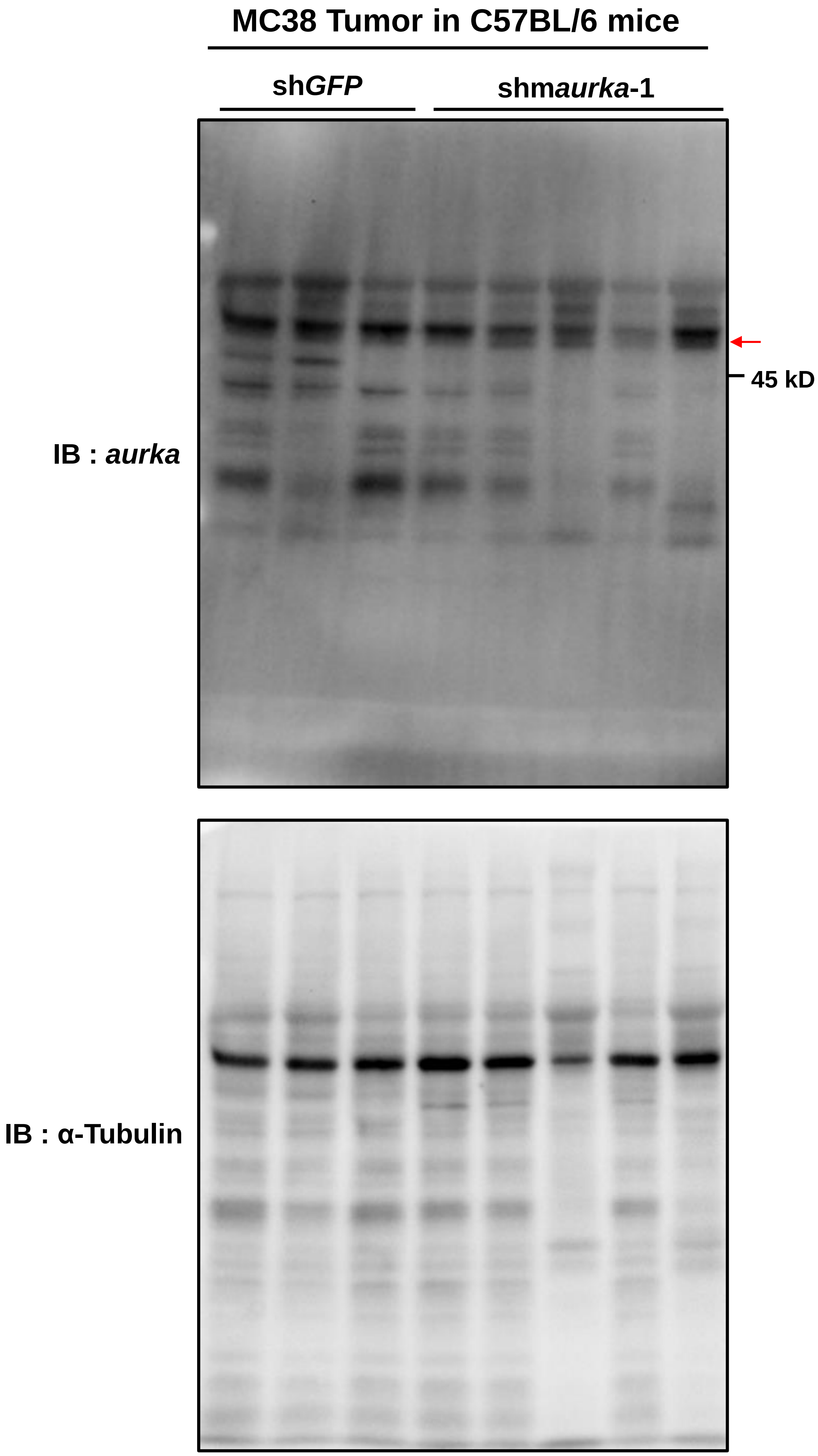

Supplementary Figure S6.

(D)

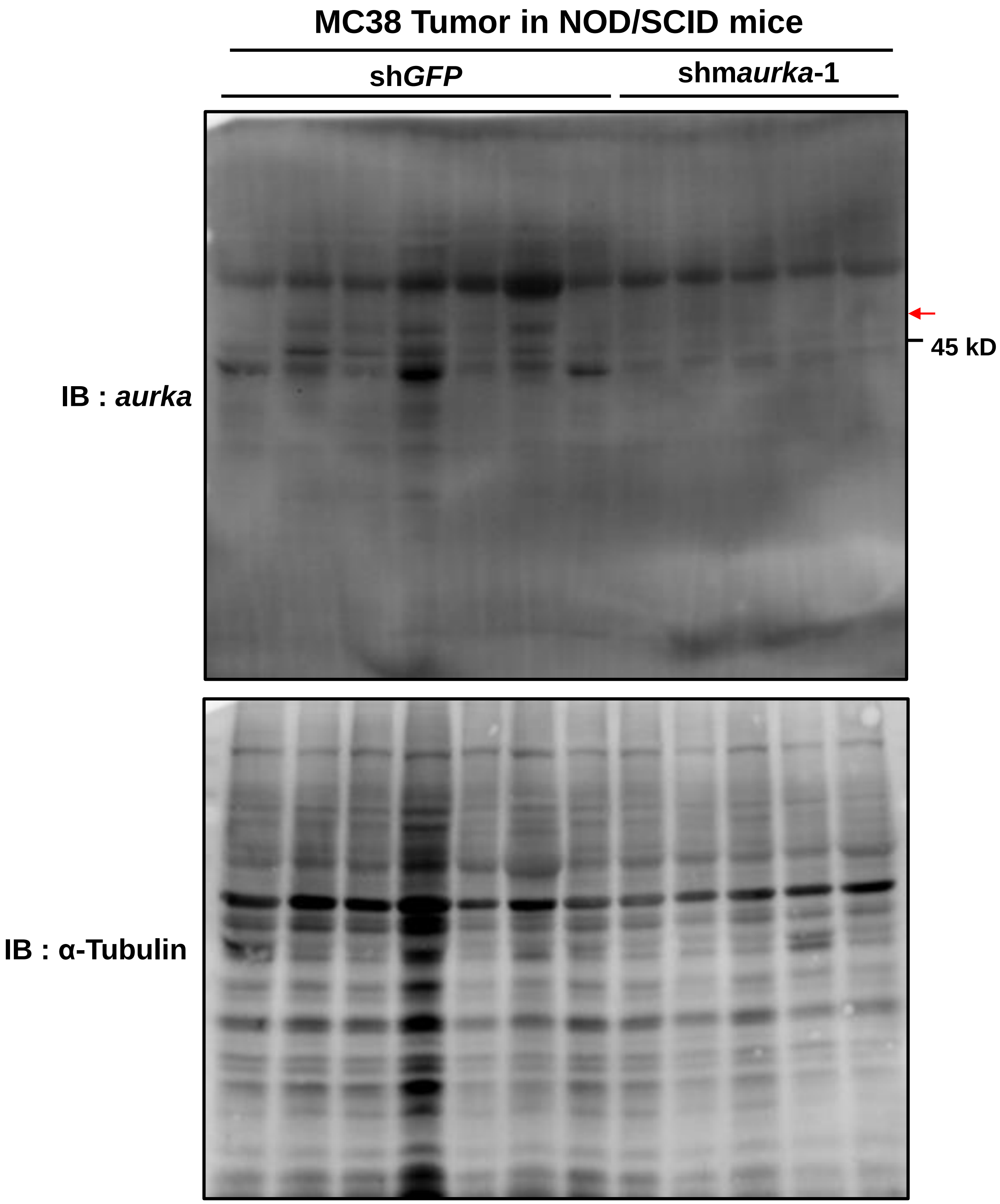

Supplementary Figure S6.

(D)

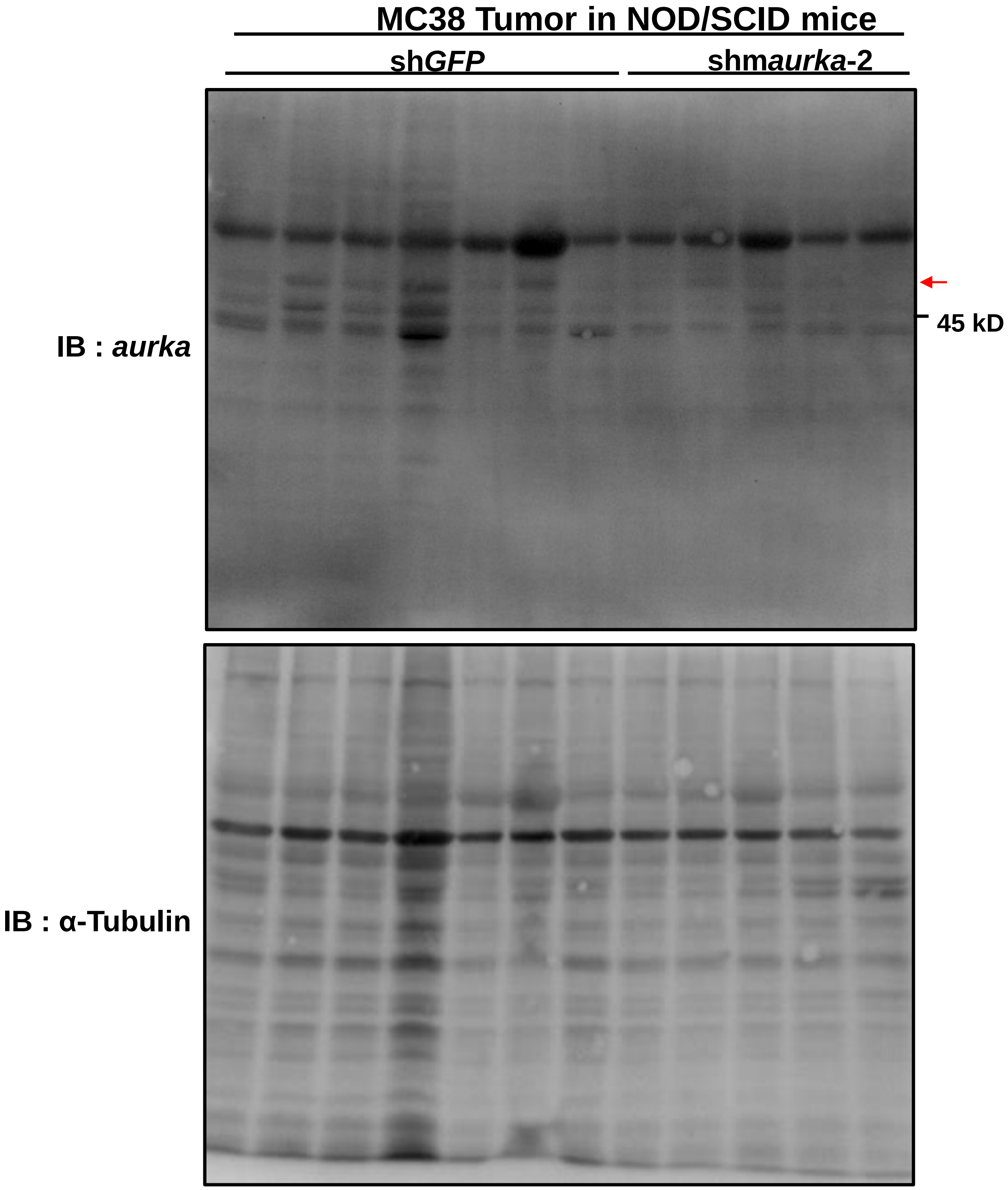

Supplementary Figure S6.

(E)

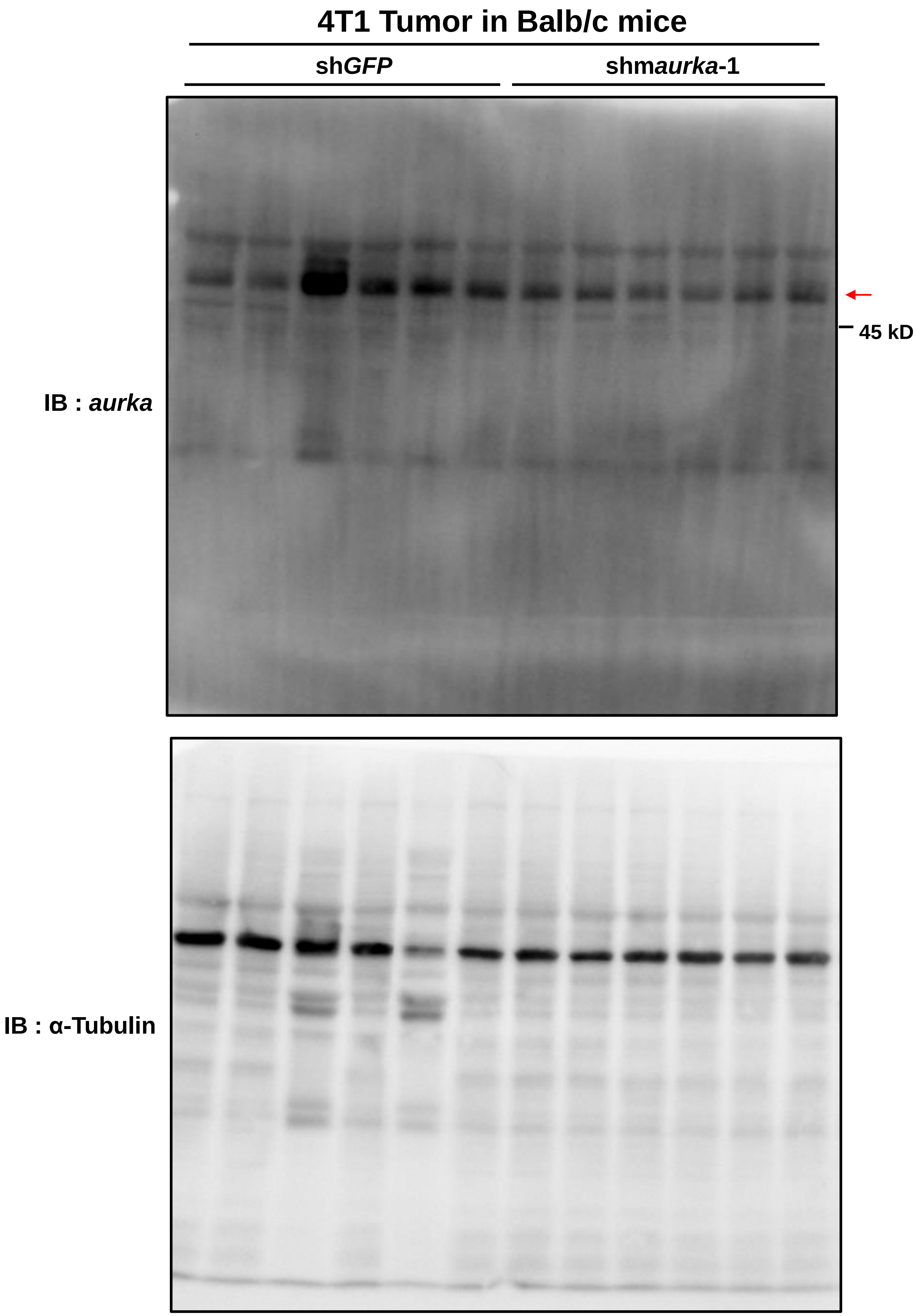

Supplementary Figure S6.

(F)

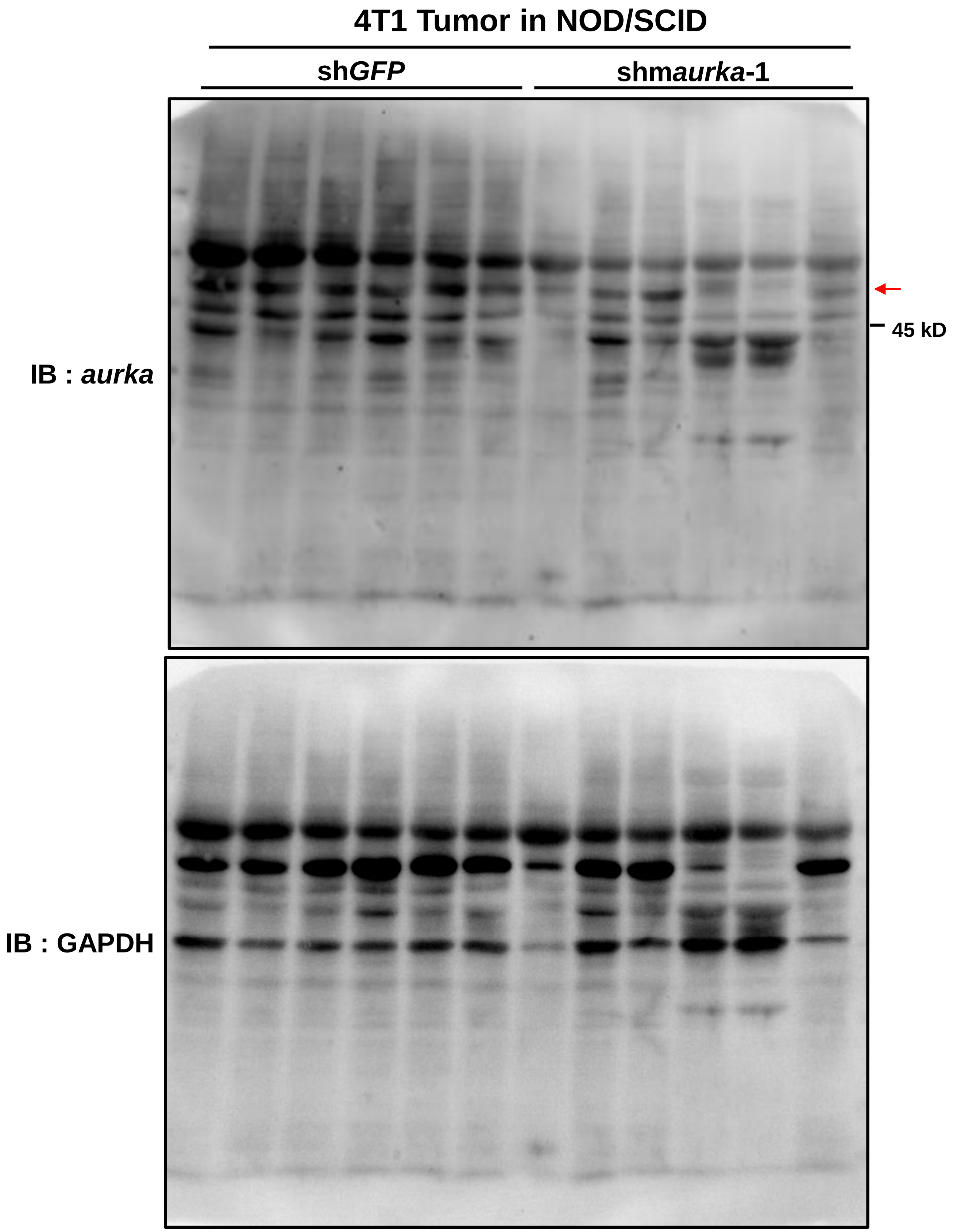

Supplementary Figure S9.

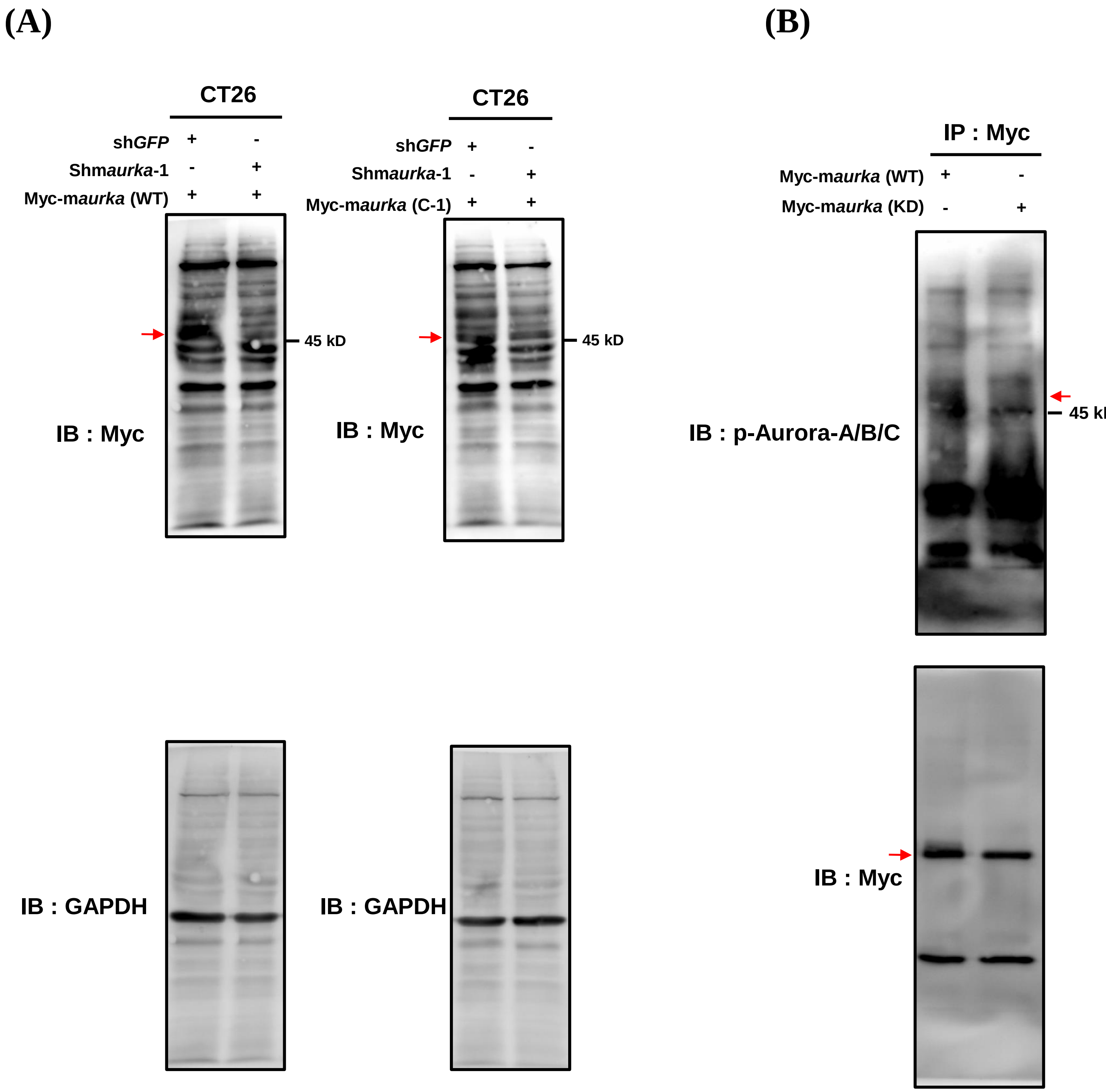

Supplement: Supplementary file 2 — Original data [file 41419_2023_6381_MOESM2_ESM.pdf]
